# Designing Single-Molecule Magnets as Drugs with Dual Anti-Inflammatory and Anti-Diabetic Effects

**DOI:** 10.3390/ijms21093146

**Published:** 2020-04-29

**Authors:** Arturo Navas, Fatin Jannus, Belén Fernández, Javier Cepeda, Marta Medina O’Donnell, Luis Díaz-Ruiz, Cristina Sánchez-González, Juan Llopis, José M. Seco, E. Rufino-Palomares, José Antonio Lupiáñez, Santiago Gómez-Ruiz, José Luis Quiles, Maurizio Battino, Duane Choquesillo-Lazarte, Ana Belén Ruiz-Muelle, Ignacio Fernández, Fernando Reyes-Zurita, Antonio Rodríguez-Diéguez

**Affiliations:** 1 Department of Inorganic Chemistry, C/ Severo Ochoa s/n, University of Granada, 18071 Granada, Spain; arturosantafe@correo.ugr.es; 2 Department of Biochemistry and Molecular Biology I, Severo Ochoa s/n, University of Granada, 18071 Granada, Spain; fatin@correo.ugr.es (F.J.); luisdiazruiz96@gmail.com (L.D.-R.); evaevae@ugr.es (E.R.-P.); jlcara@ugr.es (J.A.L.); 3 Institute of Parasitology and Biomedicine “López-Neyra”, CSIC, Av. Conocimiento s/n, 18600 Granada, Spain; 4 Department of Applied Chemistry, University of The Basque Country (UPV/EHU), 20018 San Sebastián, Spain; javier.cepeda@ehu.es (J.C.); josemanuel.seco@ehu.es (J.M.S.); 5 Department of Organic Chemistry, C/ Severo Ochoa s/n, University of Granada, 18071 Granada, Spain; mmodonnell@ugr.es; 6 Department of Physiology, University Campus of Cartuja, University of Granada, 18071 Granada, Spain; crissg@ugr.es (C.S.-G.); jllopis@ugr.es (J.L.); 7 Department of Biology and Geology, Physics and Inorganic Chemistry, Rey Juan Carlos University, Calle Tulipán s/n, 28933 Móstoles (Madrid), Spain; santiago.gomez@urjc.es; 8 Department of Physiology. Institute of Nutrition and Food Technology “Jose Mataix”, Biomedical Research Center, Avda. Conocimiento s/n, 18100 Armilla, Spain; 9 Department of Clinical Specialist and Odontostomatological Sciences (DISCO) -Sez. Biochemistry, Faculty of Medicine, Polytechnic University of Marche, 60131 Ancona, Italy; m.a.battino@univpm.it; 10 Laboratorio de Estudios Cristalográficos, IACT (CSIC-UGR), Avda. de las Palmeras 4, 18100 Armilla, Spain; duane.choquesillo@csic.es; 11 Department of Chemistry and Physics, Research Centre CIAIMBITAL, University of Almería, Ctra. Sacramento s/n, 04120 Almería, Spain; arm350@ual.es (A.B.R.-M.); ifernan@ual.es (I.F.)

**Keywords:** bumetadine, indomethacin, cobalt, coordination compound, single-ion magnet, inflammatory, diabetes

## Abstract

We have designed and synthesized two novel cobalt coordination compounds using bumetanide (bum) and indomethacin (ind) therapeutic agents. The anti-inflammatory effects of cobalt metal complexes with ind and bum were assayed in lipopolysaccharide stimulated RAW 264.7 macrophages by inhibition of nitric oxide production. Firstly, we determined the cytotoxicity and the anti-inflammatory potential of the cobalt compounds and ind and bum ligands in RAW 264.7 cells. Indomethacin-based metal complex was able to inhibit the NO production up to 35% in a concentration-dependent manner without showing cytotoxicity, showing around 6–37 times more effective than indomethacin. Cell cycle analysis showed that the inhibition of NO production was accompanied by a reversion of the differentiation processes in LPS-stimulated RAW 264.7 cells, due to a decreased of cell percentage in G0/G1 phase, with the corresponding increase in the number of cells in S phase. These two materials have mononuclear structures and show slow relaxation of magnetization. Moreover, both compounds show anti-diabetic activity with low in vitro cell toxicities. The formation of metal complexes with bioactive ligands is a new and promising strategy to find new compounds with high and enhanced biochemical properties and promises to be a field of great interest.

## 1. Introduction

The increasing consumption of sugar on our daily diet has posed diabetes as a world-wide first-line problem of which society is becoming conscious. Diabetes mellitus is a disease that manifests when the pancreas does not produce enough insulin (type I) or when the body cannot effectively use the insulin it produces (type II). Some metal ions have shown insulin-like effects by supporting the signal transduction of insulin and reducing the production of cytokines, triggering a cascade of events leading to beta-cell death during the pancreatic inflammatory process in the course of disease. Thus, there is a growing interest of the scientific community in the development of new drugs that improve diabetes treatment efficacy showing minor adverse reactions and using different administration routes. Each class of drugs has different mechanisms of action. Given that commonly used drugs are associated with several adverse effects, it is of great interest to explore new drugs that can fight this disease effectively with minimal side effects [1]. Recent studies aim to develop new drugs that can be administered orally for the treatment of diabetes [2,3]. Coordination compounds have demonstrated to inhibit enzymes that play a major role in modulating insulin sensitivity [4,5]. The design of these novel materials based on metal ions could be an excellent tool to improve the distribution and, therefore, the effectiveness of metals as oral anti-diabetic agents in the treatment of diabetes mellitus, giving an alternative to traditional insulin therapy.

In recent years, we have synthesized novel multidimensional coordination compounds, with fascinating structures and interesting physical and biological properties, by combining different transition metal ions with different organic ligands, such as triazolopyrimidine derivative ligands [6,7] and nitrogen ligands with carboxylate groups [8]. More specifically, in the field of diabetes, we have designed Zn- and V-based compounds [9,10] with potential application in the treatment of this disease, a pathology in which Zn compounds have shown promising hypoglycemic properties [11]. Other materials based on different metal ions, such as vanadium compounds, have shown promising activity as hypoglycemic agents for the pharmacotherapy of diabetes [12].

In a parallel manner, the use of inorganic medicinal chemistry has been substantially increased over the past few decades, being focused on the development of metal complexes for the treatment of several diseases, such as cancer, inflammatory, or autoimmune diseases [13]. More interestingly, there are studies on the benefits of anti-diabetic agents with anti-inflammatory properties. In particular, recent data suggest that immunomodulatory treatments may have beneficial effects on glycemia, b-cell function, and insulin resistance [14]. Therefore, developing of a drug with insulinomimetic-anti-inflammatory duality is undoubtedly a great advance to face up to diabetes disease.

During inflammation process, different molecular routes and a wide variety of protein kinases are activated, as MAPK (mitogen-activated protein kinases), JAK (Janus-activated kinases), PI3K/AKT (phosphatidylinositol-3-kinase). As a consequence, several transcription factors related to the proliferation and inflammation routes are activated in response to these protein kinases, for example, STAT (signal transducer activator of transcription), NF-kB (nuclear factor kappa B), activation proteins 1 (AP-1), or HIF-1 (hypoxia inducible factor 1-α), leading to the expression of pro-inflammatory genes that encode pro-inflammatory enzymes as iNOS (nitric oxide synthase inducible) and COX-2 (cyclooxyenase-2), responsible for the synthesis of eicosanoids and other proteins mediators, as cytokines and interleukins. Disturbing the activation of these transcription factors can produce aberrant cell growth, carcinogenic cell transformation, angiogenesis, and metastasis.

Nitric oxide (NO) is a very important mediator in acute or chronic inflammation. This nitrogen radical is produced by constitutive and inducible nitric oxide synthases. The inducible enzyme is activated in response to pro-inflammatory signals, such as LPS (bacterial lipopolysaccharide), enterotoxin, or cytokines as TNFα (tumor necrosis factor α) or INF- γ (interferon-γ) in macrophages, hepatocytes, and endothelial cells. The reduction of NO can be produced by direct scavenger action of NO Radicals, iNOS inhibition enzyme activity, and/or iNOS gene expression inhibition. In this study, the inhibition of NO production in activated LPS RAW macrophage cells have been used as an indirect marker of the inflammatory process.

**Scheme 1:**
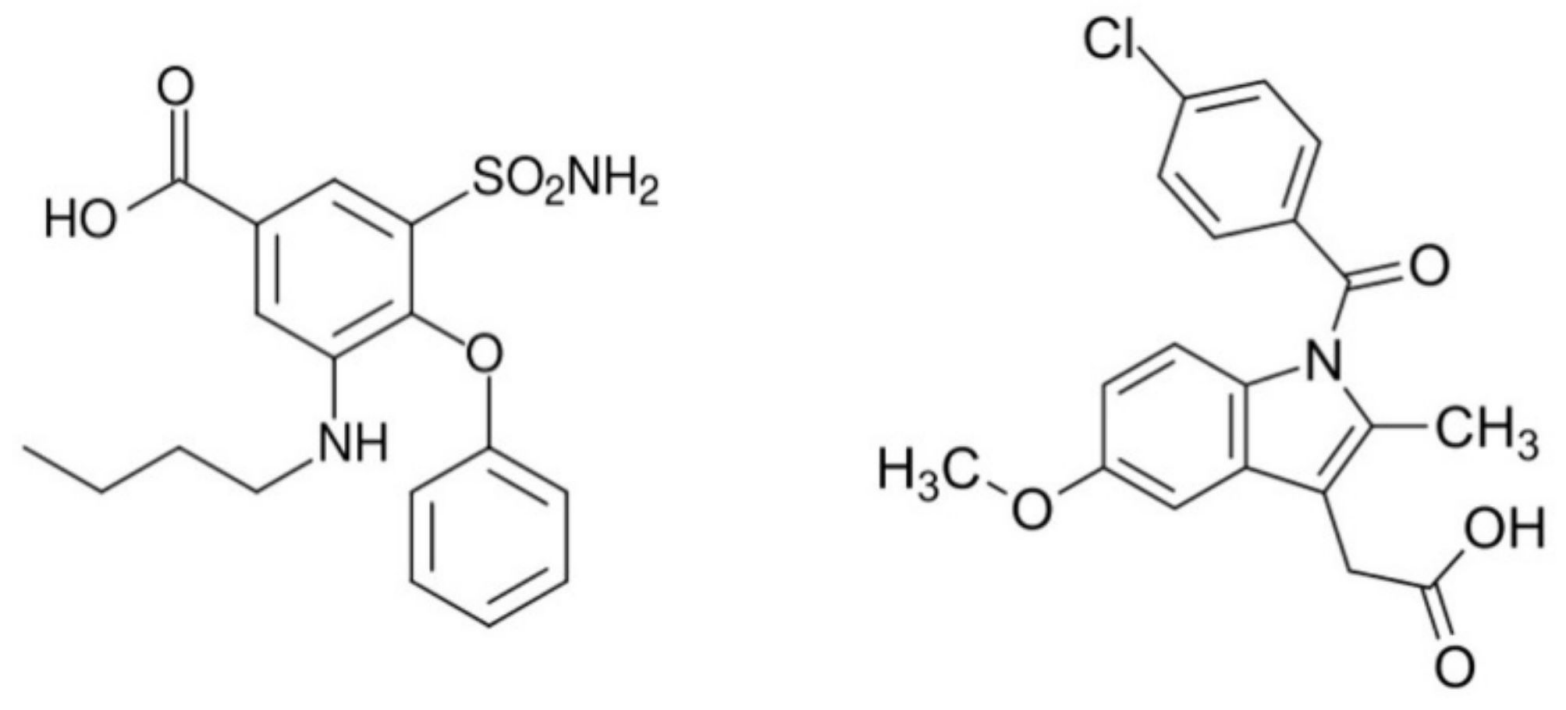
Bumetanide (left) and indomethacin (right) organic ligands used as drugs to construct coordination compounds based on cobalt.

To construct novel materials, in this case, we have chosen bumetanide (bum) and indomethacin (ind) anti-inflammatory agents (Scheme 1).

Indomethacin (1-(p-chlorobenzoyl)-5-methoxy-2-methylindole-3 acetic acid) is a nonsteroidal anti-inflammatory drug (NSAID) with very effective antipyretic, analgesic, and anti-inflammatory activity. Its pharmacological effects consist of an inhibition of cyclooxygenase enzyme (COX), reducing the synthesis of prostaglandins. Especially, ind inhibits the catalytic activity of the COX enzymes (isoforms COX-1 and COX-2), producing the inhibition of prostaglandins synthesis via the arachinodonic acid pathway, causing a reduction of pain, fever, and inflammation [15] due to their ability to reduce blood flow, modulating nitric oxide pathways and vasoconstriction [16]. Ind is more powerful than other NSAIDs commonly used as anti-inflammatory compounds, such as ibuprofen or aspirin [17].

Bum is a potent loop diuretic effective in the treatment of the oedema associated with different inflammatory process, as congestive heart failure, hepatic and renal diseases, acute pulmonary congestion, premenstrual syndrome, and diuresis during and after surgery. In relation to the inflammatory process, bum has been associated with the Na^+^-K^+^-2Cl^−^ co-transporter BSC2 (NKCC1), which is regulated by inflammatory cytokine stimuli. This co-transporter is responsible for maintenance of a selective permeability barrier in mammalian cells [18]. Bum is well known for its ability to inhibit NKCC1 chloride-importer inhibitor [19]. However, bum inhibits the trans-membrane conductance regulator CFTR GCI [20]. NKCC1 and CTFR are both involved in a variety of biological processes ranging from the regulation of macrophage activation to the modulation of cytokine production.

Accordingly, we decided to work with these organic molecules as ligands, ind and bum, to form coordination complexes due to the fact very few examples are reported so far. In this way, we could offer to the scientific community multifunctional materials with different biomedical applications. In particular, a perusal of the CCDC database shows that only the sodium and potassium salts of bum ligand have been characterized [21]. On the other hand, only some examples of coordination compounds based on copper [22], tin [23], and zinc [24] have been found for the ind molecule. All the above-mentioned leads us to the conclusion that the biological application of coordination compounds based on these therapeutic agents is almost unexplored.

In order to obtain single-molecule magnets (SMMs) with biological properties, we chose cobalt due to this metal ion generally exhibiting large magnetic moments and high anisotropy. On the one hand, the fact that cobalt has such a predictable octahedral coordination sphere could help us control the structure of the mononuclear compound. In this sense, ind and bum are adequate because, possessing a single carboxylate group, they may coordinate occupying two coordination positions in the cobalt sphere. Being monoanionic ligands, coordination of two ligands by a cobalt center would balance the charge to yield neutral cobalt-based monomeric complexes. Moreover, thanks to the large size of the therapeutic agents, cobalt ions could be separated away in the crystalline structure to obtain novel materials exhibiting interesting magnetic properties provided that they maintain a large anisotropy, using either transition metal ions with first-order orbital angular momentum [25], such as Co^II^ and Fe^II^, or very anisotropic lanthanide ions, such as Dy^III^ [26]. Though these interesting magnets are being mainly suggested for their applications in molecular spintronics and ultrahigh density magnetic recording [27], their utility could also be extended to research in the field of biomedicine.

For all the above reasons, the milestone that we propose here was to synthesize novel cobalt mononuclear coordination compounds with potential biomedical activities that, at the same time, exhibit slow relaxation of magnetization. In this paper, we present two novel mononuclear coordination compounds, [Co(bum)_2_(H_2_O)_2_](H_2_O)_2_ (**1**) and [Co(ind)_2_(EtOH)_2_] (**3**) (Hbum = bumetanide (**2**) and Hind = indomethacin (**4**)), synthesized using conventional routes.

## 2. Results and Discussion

### 2.1. Description of the Structures

#### 2.1.1. Structural Description of [Co(bum)_2_(H_2_O)_2_](H_2_O)_2_ (1)

**Figure 1:**
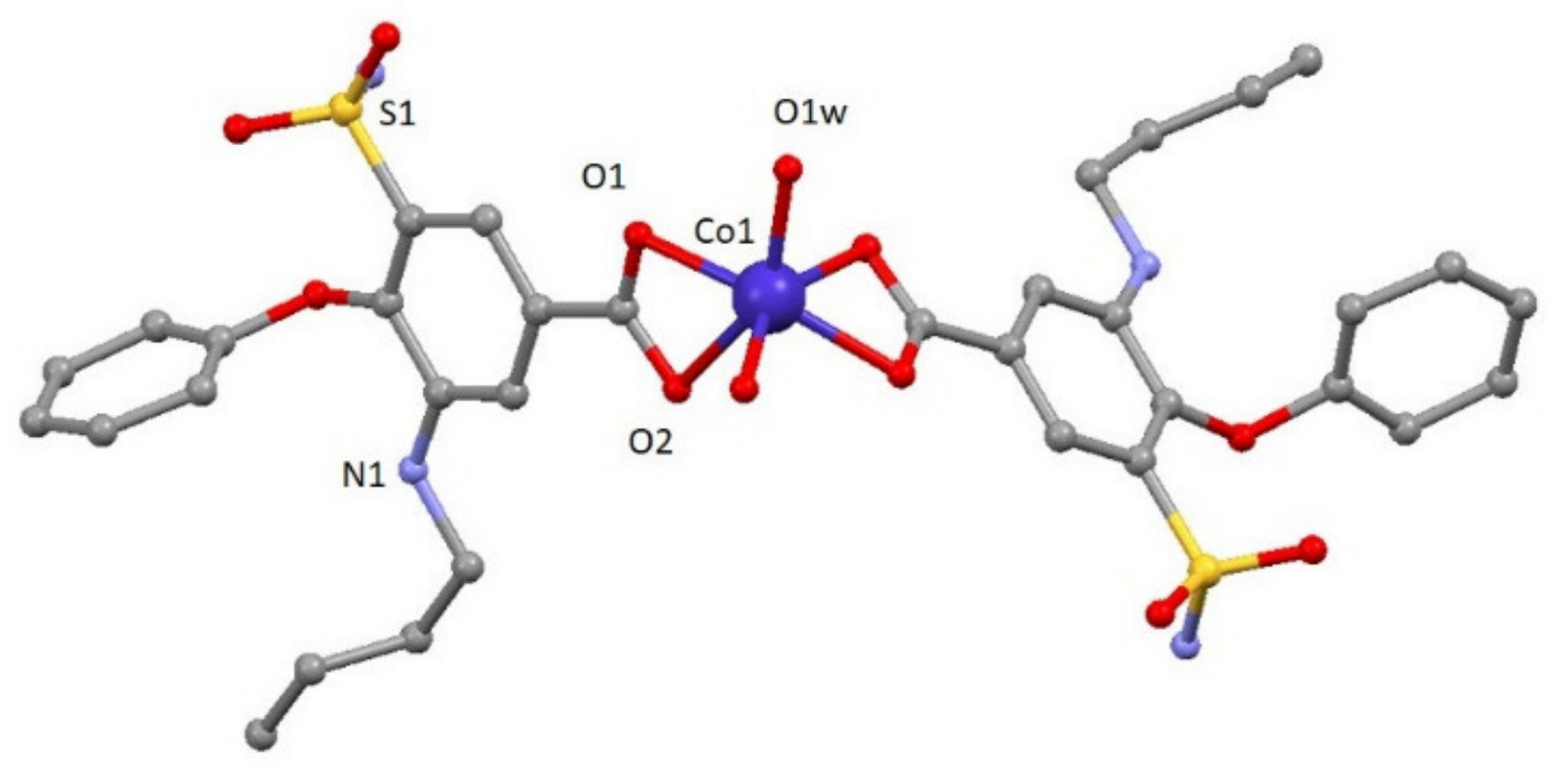
A perspective view on the molecular structure of [Co(bum)_2_(H_2_O)_2_].(H_2_O)_2_ (**1**). Crystallization water molecule and hydrogen atoms have been omitted for clarity.

Compound **1** crystallizes in the monoclinic *P*2/*c* space group. A perspective view on the molecular structure of **1** is shown in Figure 1. Selected bond lengths are given in Table S1. The structure consists of one mononuclear [Co(bum)_2_(H_2_O)_2_] neutral complex and two crystallization water molecules. Each Co(II) ion shows a distorted octahedral CoO_6_ stereochemistry formed by two oxygen atoms in *cis* positions belonging to two different water molecules and four oxygen atoms of carboxylate groups pertaining to bumetanide ligands. Continuous shape measures confirm the large distortion of the environment compared to an ideal octahedron (S_OC_ = 5.89, see Table S3). The two Co–O_bum_ bond distances have values of 2.096(3) and 2.233(3) Å, whereas Co–O_w_ distance is 2.025(3) Å. The *cis* and *trans* angles vary in the 60.51(13)–113.86(14)° and 147.7(2)–148.33(13)° ranges, respectively. It should be noted that the most important parameter bringing the distortion of octahedral coordination environment is due to the acute O1-Co-O2 bite angle of the carboxylate group (60.51(13)°). This compound shows a complex and beautiful hydrogen bonding network, in which O1W, O2W, O1-O5 and N2 atoms are involved. These hydrogen bonds generate a 2D network with distances in the 2.630(5)–3.011(6) Å range, among which that established in O1w-H1WA∙∙∙O2 (2.630(5) Å) must be highlighted because it is responsible for the relative coplanar disposition of the two coordinated bumetanide ligands. This 2D hydrogen network can be described by anti-parallel chains constructed of these mononuclear entities (Figure S3). Selected bond distances and hydrogen bonds are given in Table S1 and S2, respectively.

To the best of our knowledge, it is the first time that a coordination compound with bumetanide ligand has been synthesized.

#### 2.1.2. Structural Description of [Co(ind)_2_(EtOH)_2_] (3)

**Figure 2:**
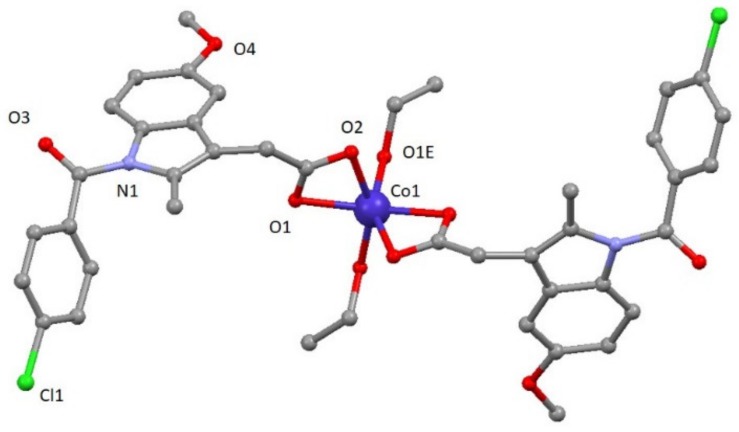
A perspective view on the molecular structure of [Co(ind)_2_(EtOH)_2_] (**3**). Hydrogen atoms have been omitted for clarity.

Compound **3** crystallizes in the monoclinic *C*2/*c* space group. A perspective view on the molecular structure of **3** is shown in Figure 2. Selected bond lengths are given in Table S2. The structure of [Co(ind)_2_(EtOH)_2_] (**3**) consists of mononuclear compounds of cobalt with two ligands and two coordinated solvent molecules (Figure 2). The asymmetric unit contains one cobalt atom, one ind ligand, and one ethanol molecule. In the structure, each Co^II^ atom is located on an inversion center and exhibits a distorted octahedral CoO_6_ geometry established from four oxygen atoms of two carboxylate groups pertaining to indomethacin ligands and two oxygen atoms from two different ethanol molecules. Co^II^–O_ind_ bond distances have values of 2.110(2) and 2.185(2) Å, whereas Co^II^–O_ethanol_ distance is 2.025(3) Å. The *cis* and *trans* angles vary in the 61.15(8)–104.51(9)° and 152.04(12)–154.90(8)° ranges, respectively. As a result, the distortion of the coordination environment with regard to an ideal octahedron is lower than for **1** but still significant (S_OC_ = 4.48, see Table S3).

This compound shows a crystalline network that can be described by chains constructed of these mononuclear entities highlighting the O1E-H1E∙∙∙O2 hydrogen bond with a value of 2.606(3) Å. Selected bond distances are given in Table S3.

### 2.2. Magnetic Properties

**Figure 3:**
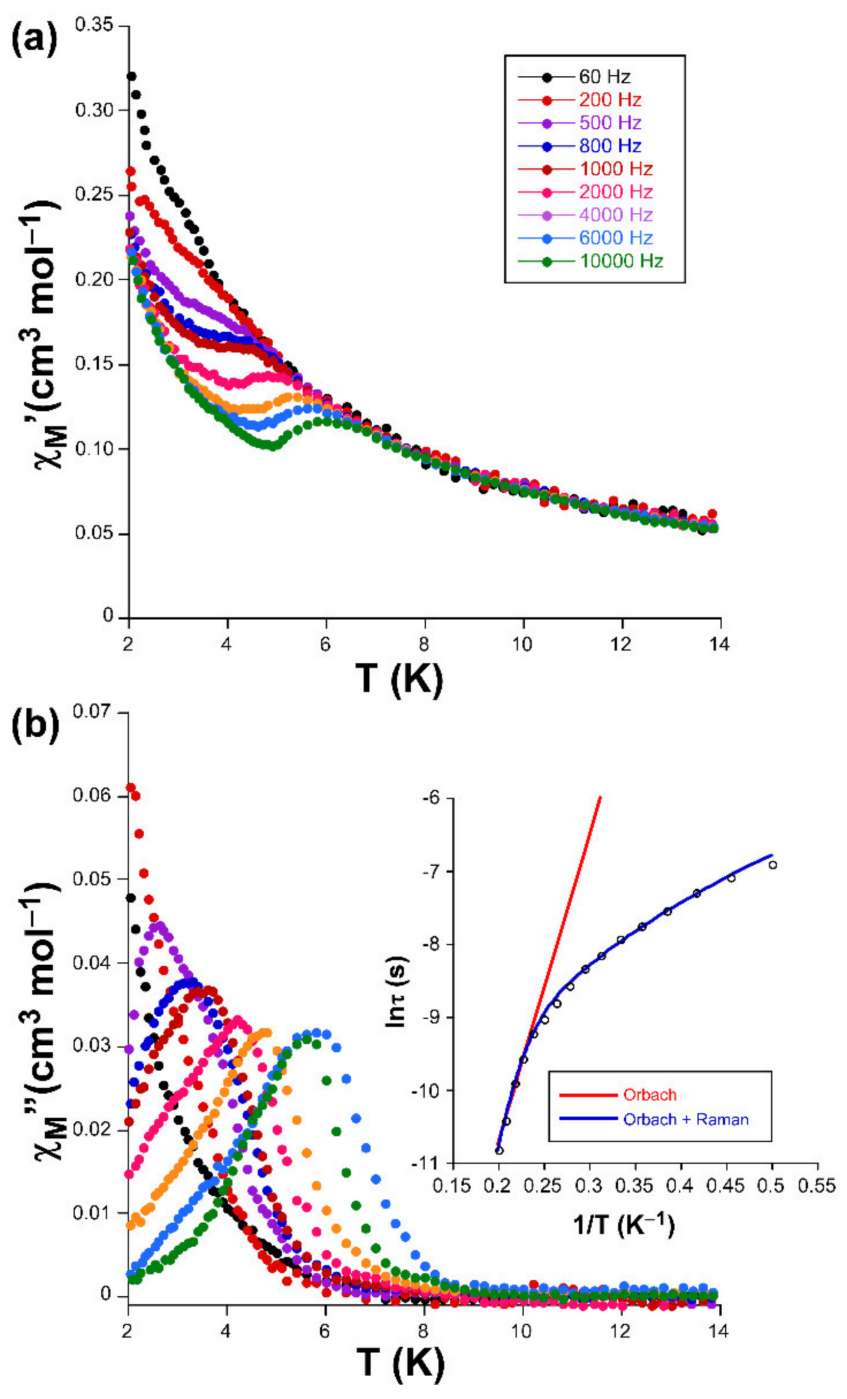
Temperature dependence of the (**a**) *χ_M_’* and (**b**) *χ_M_’’* signals for compound **1** together with the Arrhenius plot of the relaxation times.

**Figure 4:**
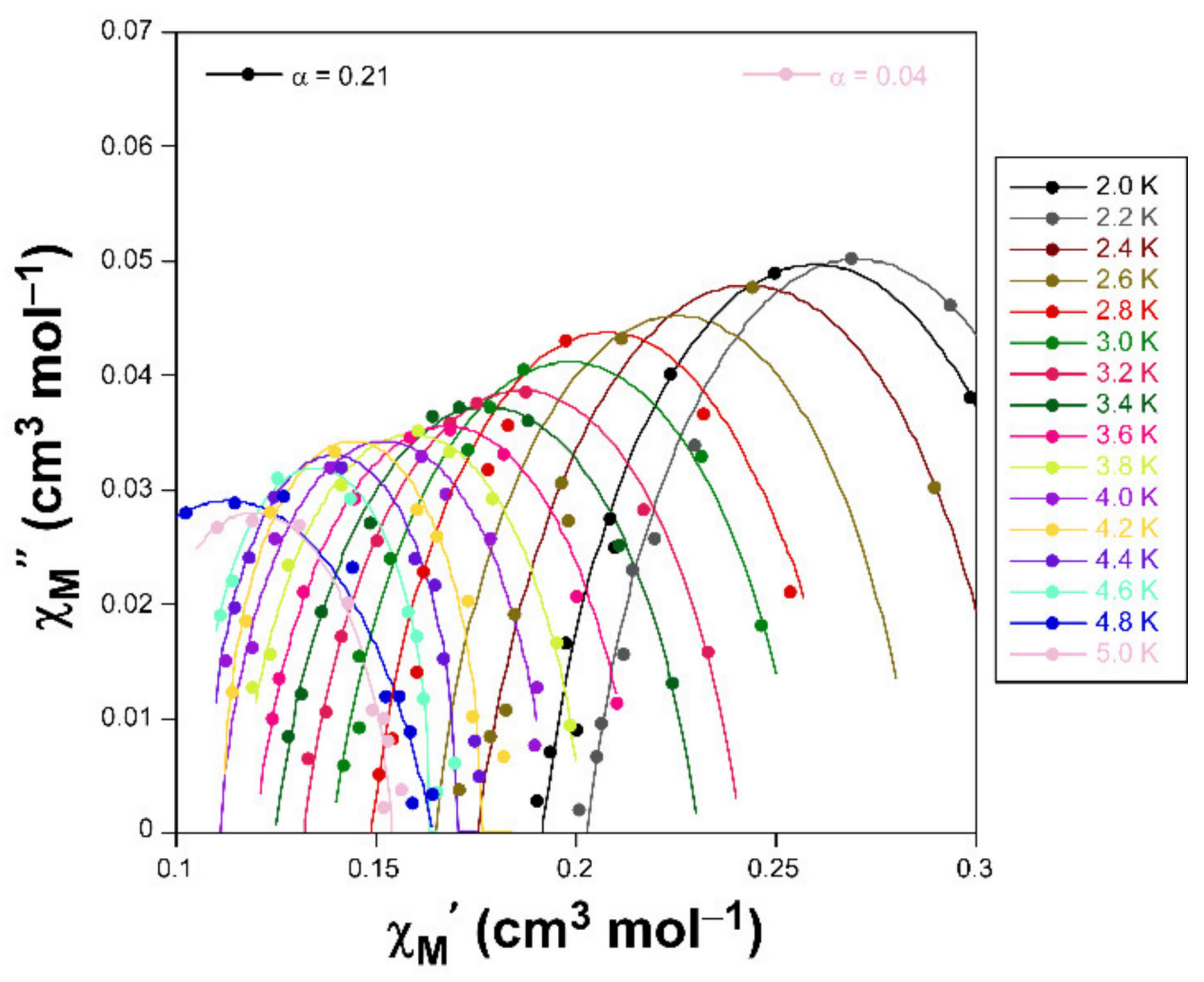
Cole-Cole plots in the form of *χ_M_’’* vs *χ_M_’* for compound **1** under an applied field of 1 KOe.

**Figure 5:**
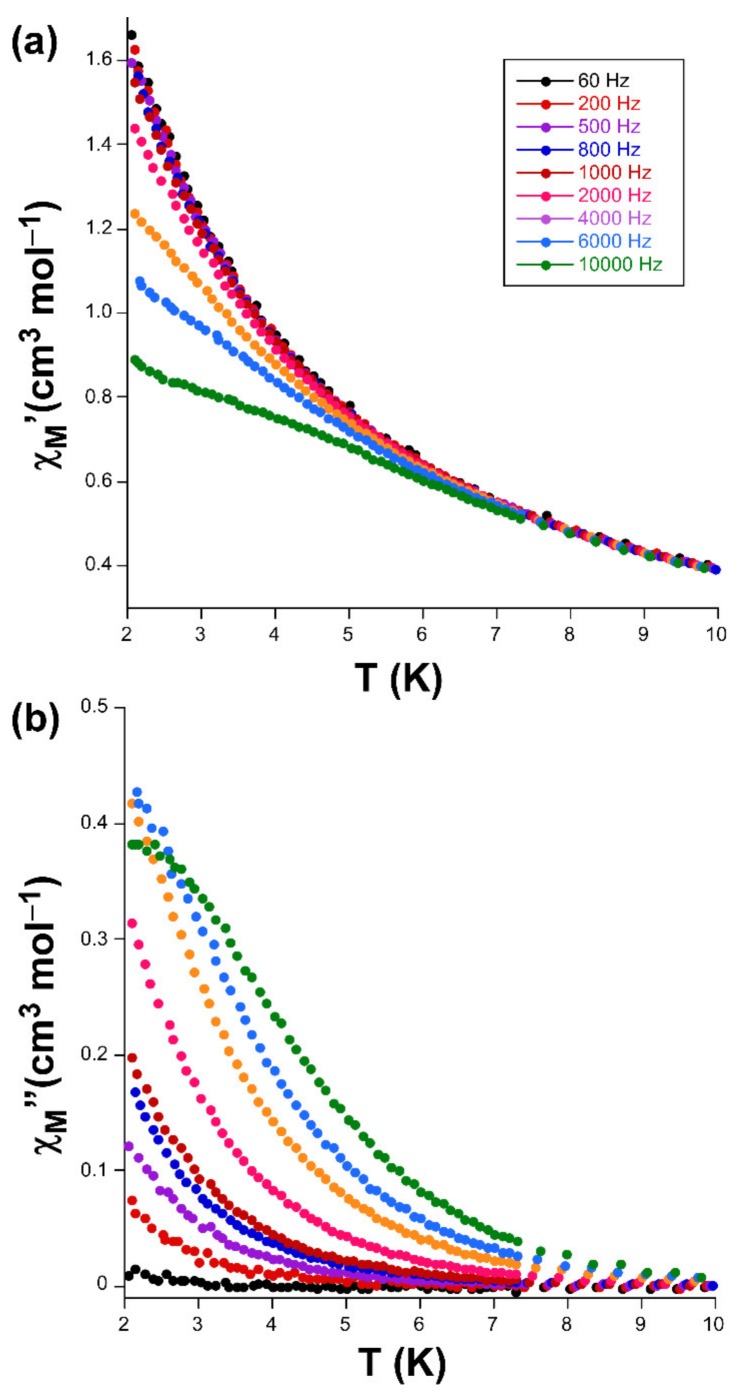
(**a**) *χ_M_’* vs T and (**b**) *χ_M_’’* vs T plots signals for compound **3**.

Alternating-current (*ac*) susceptibility measurements under an AC field of 3.5 Oe were performed in order to study the spin dynamics of these cobalt-based compounds in view of the isolated nature of the spin carriers in their crystal structures (closest Co···Co separations are of ca. 5.39 and 5.27 Å mediated by intermolecular hydrogen bonds). As it is well-known, small distortions introduced in the coordination environments of high-spin octahedral Co(II) centers break the degeneracy of the ^4^T_1g_ ground state [28], causing substantial zero-field splitting (zfs) that plays a very important role in the SIM behavior [29]. On accounts of previously reported magnetization measurements and ab initio calculations, large values of zfs parameters are expected irrespective of their sign (leading to easy-axis or easy-plane anisotropy) for distorted octahedral environments such as those present on these compounds (as shown by CShMs: *S_OC_* = 5.89 and 4.48 for **1** and **3**, respectively) [30]. However, no frequency dependent signals were observed above 2 K in the absence of an applied external DC field, which is probably related to the presence of fast quantum tunneling of the magnetization (QTM) derived from intramolecular and/or strong hyperfine interactions with the I = 7/2 nuclear spin of the Co(II) atom [31,32]. Instead, QTM is effectively suppressed by applying a DC field of 1000 Oe, in such a way that both compounds exhibit SIM behavior with characteristic temperature-dependent in-phase (*χ_M_’*) and out-of-phase (*χ_M_’’*) signals [33,34]. In particular, **1** presents the maxima in the *χ_M_’’* signal for frequencies larger than 500 Hz (Figure 3), which allow performing further analysis (Figure 4). However, maxima of compound **3** occur at too low a temperature as to be observed (see Figure 5).

Accordingly, Cole-Cole plots represented in the temperature region delimiting these maxima (2–5 K range) show a well-defined semi-circular distribution that could be successfully fitted by means of the Debye model (Figure 4) [35]. The resulting values of *α* range between 0.21 and 0.04, a wide dispersion that suggests the presence of more than one mechanism involved in the spin relaxation of **1**. The distribution of the relaxation times in the Arrhenius plot (expressed as ln(*τ*) vs 1/*T*, where*τ*is extracted from the fit of *χ_M_’’* at each temperature) confirm the previous statement by showing a curvilinear shape.

In any case, taking into account high-temperature range that follows the Arrhenius law, an energy barrier for the reversal of the magnetization (*U_eff_*) of 42.3 K and *τ*_0_ of 4.53 × 10^–9^ s are estimated through the thermally activated Orbach process (Equation (1), see inset of Figure 3).The value of *U_eff_* and *τ*_0_ fall within the typical range found for SIMs based on octahedral Co(II) complexes [36,37].

*τ* = *τ*_0_ exp(*U_eff_*/*κ_B_T*) (1)

This kind of Arrhenius representation clearly deviating from linearity is commonly related to the presence of additional relaxation mechanisms (such as filed-induced phonon bottleneck direct relaxation, Raman relaxation and/or QTM), in good agreement with aforementioned *α* values [38]. Among these possible mechanisms, QTM was initially discarded given the absence of rapid increase in the *χ*_M_’’ curves at low temperature (below the maxima). In the direct and Raman processes, relaxation follows *τ*^–1^ = *CT^n^* equation, where *n* should equal 1 and 9 for Kramers ions, respectively, for direct and Raman processes, although values in the 1–6 range are acceptable in practice provided that the relaxation takes place through phonons. Attempts to fit the relaxation times, though reliably reproducing the experimental curve, gave rise to a diversity of results, most of which lack physical sense. Therefore, only Orbach and Raman were considered for the fitting process, making use of the expressions gathered in Equation (2):

*τ*^−1^ = *B_Raman_T^n^* + *τ*_0_^−1^ exp(*−U_eff_*/*κ_B_T*) (2)

Results of the best fit are *B_Raman_* = 115(4) s^–1^, *n* = 2.9(3), *τ*_0_ = 1.69 × 10^–11^ s and *U_eff_* = 71(4) K, which can be considered as relatively high compared to those previously reported for Co^II^ based SIMs [39,40].

### 2.3. Anti-Diabetic Properties

The nematode *Caenorhabditis elegans* has proven to be an important animal model to study the molecular mechanisms of drug effects and disease pathogenesis and for that, it has been used extensively as a model organism since 1974. Many key findings with relevance for mammals were discovered in this well-characterized model organism, it became first organism which was completely sequenced, showed a strong conservation of biological principles between *C. elegans* and mammals with approx. 60–80% of gene homologues between human and *C. elegans* [41]. This model has been used extensively to study various diseases, including diabetes, an endocrine dysfunction resulting from insulin deficiency or incapability of peripheral tissues to respond to insulin [42].

**Figure 6:**
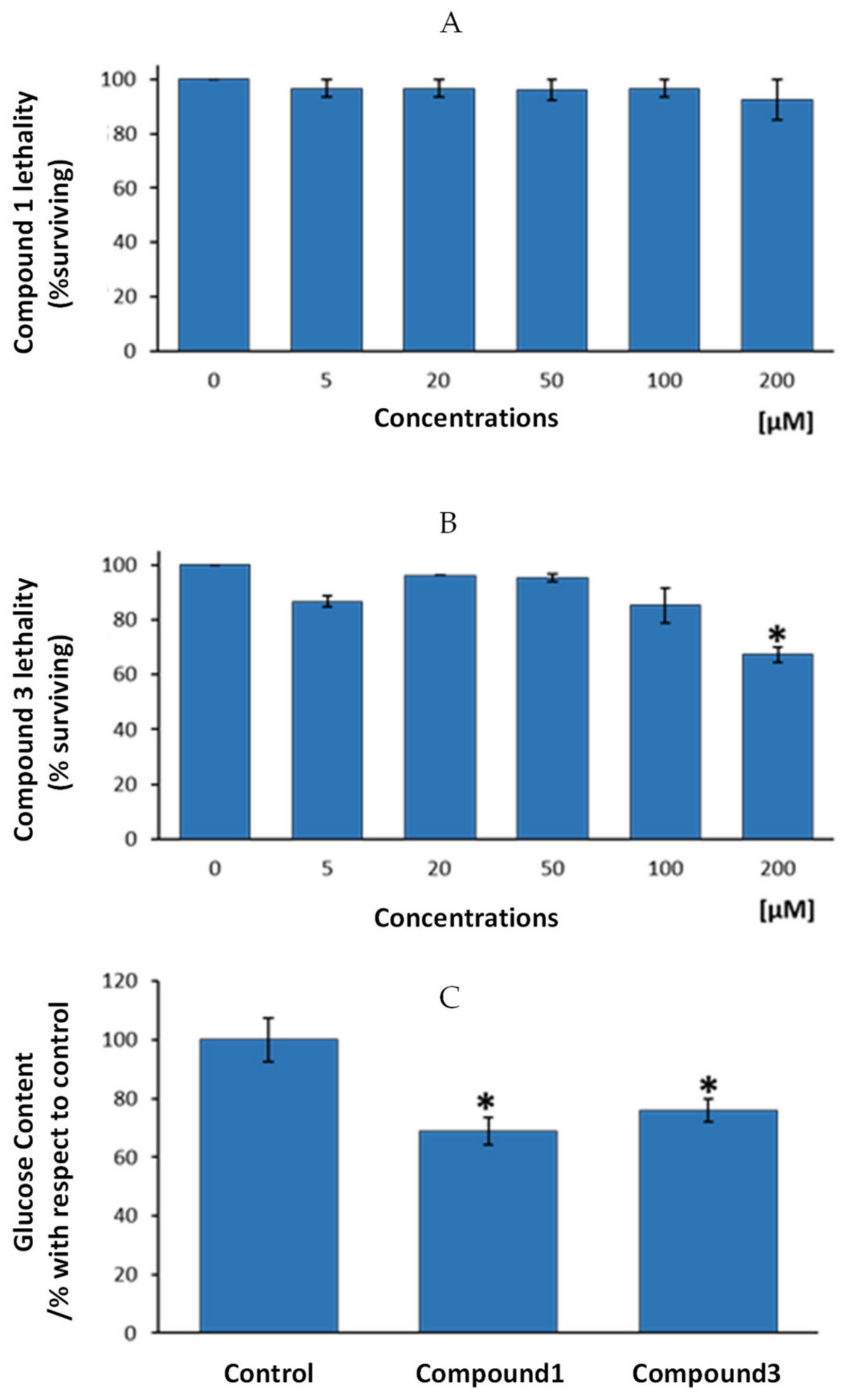
Study of the anti-diabetic properties of compounds **1** and **3** in the nematode *C. elegans*. (**A**) Lethality analysis for compound **1**. (**B**) Lethality analysis for compound **3**. (**C**) Changes in glucose content in the nematodes after exposure of 100 µM of compounds **1** and **3**, respectively. Results represent the mean ± SEM of three independent experiments. * Statistically significant differences with respect to 0 µM dosage (**B**) or to the control group (**C**).

Results from the present study show that exposure of nematodes to increasing concentrations of compound **1** resulted in a lack of acute toxicity, expressed by the lethality test, up to a dosage of 200 µM (Figure 6A). For compound **3**, a statistically significant lethality level was only showed for 200 µM, the highest tested dosage (Figure 6B).

According to these results, the authors decided to test anti-diabetic potential of assayed compounds (compound 1 and 3) by using the highest and non-toxic dosages for any of the two compounds, i.e., 100 µM dosage. Figure 6C presents glucose levels in the nematodes after four days exposure to 100 µM of compounds **1**, **3** or to control vehicle, bum (compound **2**) and ind (compound **4**). Results showed that compound **1** presented statistically significant lower glucose levels that control group (mean reduction of 31%, *p* < 0.05). In the same way, compound **3** exhibited lower glucose than nematodes from the control group (24% less glucose, *p* < 0.05). According to these results, we can conclude that both, compounds **1** and **3** might be endowed with anti-diabetic properties, since one of the main features of diabetes is an excess of glucose in the organism, irrespective of the cause of this excess (diminished insulin production or enhanced diabetes resistance). Cobalt usefulness in the management of diabetes and glycemic control in streptozotocin-diabetic rats has been previously reported [43], displaying those cobalt-treated diabetic rats demonstrated an enhanced ability to clear a glucose overload compared to the untreated control group. However, as far as we know, this is the first study based on cobalt-based compounds on glucose control in *C. elegans*. Thus, the present research demonstrates, on one hand, that the newly synthetized compounds might be of interest in the management of diabetes. On the other hand, it is corroborated here that the potential of the use of a high-throughput screening model like *C. elegans* to test cobalt-based newly designed compounds for the putative treatment of altered glucose metabolism-dependent diseases.

### 2.4. Raw 264.7 Cell Viability

Traditionally, small organic molecules have been described with anti-cancer and anti-inflammatory properties useful as drugs [44,45]. Currently, has been reported that metal complexes possess remarkable advantages that render them as attractive alternatives to small organic molecules for the development of potential therapeutic agents, among others they show higher half-lives to allow their preferential tissue accumulation, due to their highest vascular permeability that permits their retention on tumor tissues [46].

The coordination of bioactive molecules to metal ions is a common strategy to improve the therapeutic potency and/or to reduce the toxicity of drugs molecules, giving their new properties as increased lipophilicity profiles compared to the free ligands, allowing to pass through cell membranes more easily. Additionally, the complexation of nonsteroidal anti-inflammatory drugs (NSAIDs) may be an effective strategy to reduce the adverse side effects of these agents [47].

**Figure 7:**
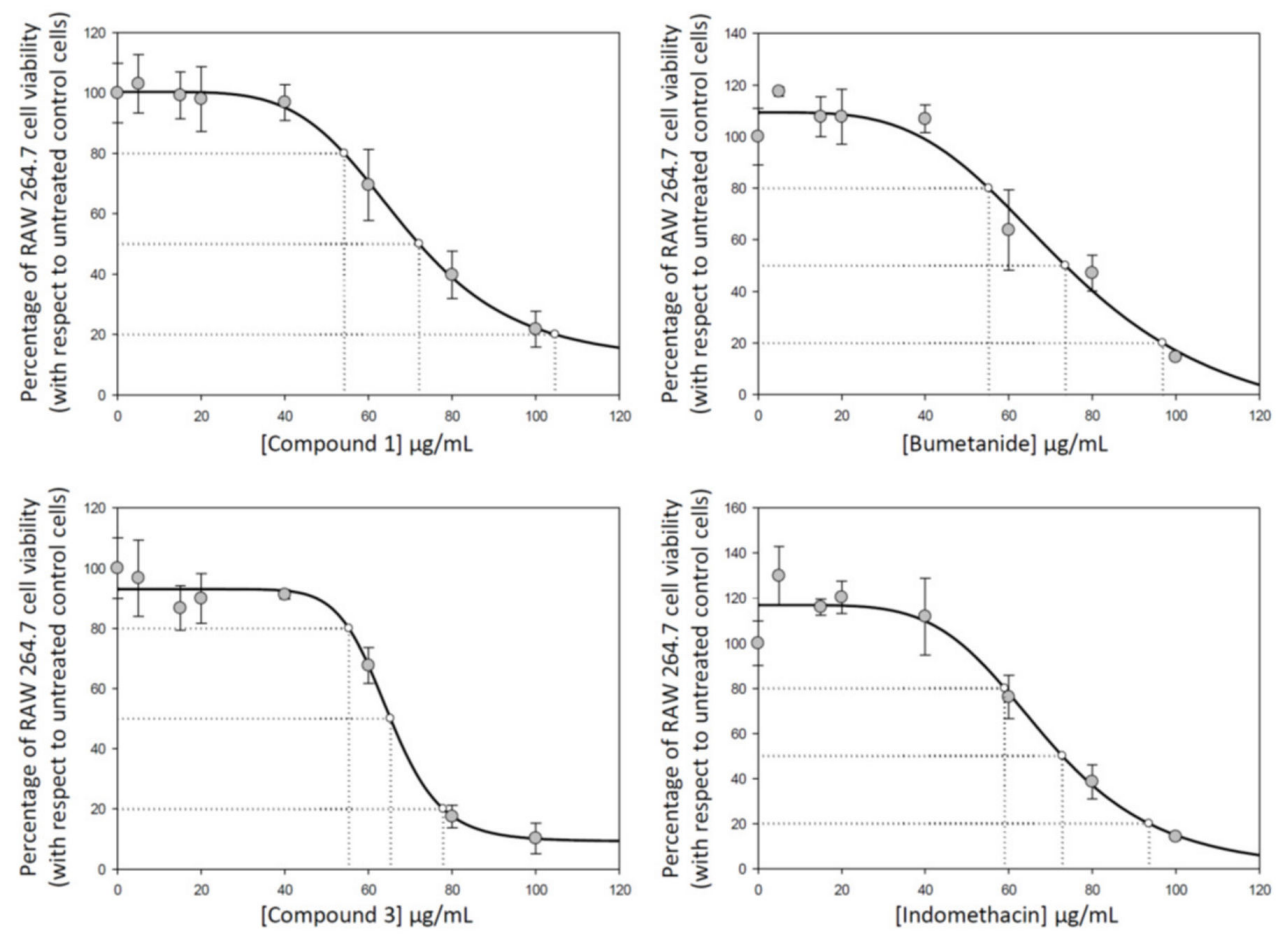
Effect of compounds **1**, **2**, **3**, and **4**, on cell proliferation in RAW 264.7 macrophage murine cells, after 72 h of treatment with assayed compounds in a wide range of concentration from 0 to 100 μg/mL. Each point represents the mean value ± S.D. of at least two independent experiments performed in triplicate. IC_20_, IC_50_, and IC_80_ are the concentrations required for growth inhibition of 20%, 50% and 80%.

Cytotoxicity of compounds **1**, **2**, **3**, and **4** were determined on RAW 264.7 murine macrophage cells to establish infra-cytotoxic work concentrations and to assure that antiflammatory effects were due to an inflammatory process and not due to their cytotoxicity. The cytotoxicity range was determined by the well-established MTT assay, where (3-(4,5-dimethylthiazol-2-yl)-2,5-diphenyltetrazolium bromide) is transformed to formazan into peroxisomes from viable cells, while dead cells lose the ability and therefore show no signal. IC_50_, the total concentration of inhibitor required to reach 50% inhibition, were calculated by nonlinear regression taking the concentration required to bring the curve down to point half way between the top and bottom plateaus of the curve (using the SigmaPlot program). The results showed similar cytotoxicity values for all compounds under study in the conditions assayed (from 0 to 100 μg/mL) (Figure 7). Concretely, IC_50_ concentrations (50% inhibition) were 72.10 ± 6.63 μg/mL for compound **1**, 71.74 ± 9.64 μg/mL for compound **2**, 65.27 ± 1.41 μg/mL for compound **3**, and 72.55 ± 4.12 μg/mL for compound **4** (Figure 7). Moreover, we determine the compound concentrations required for 20% and 80% of growth inhibition (IC_20_ and IC_80_) to analyze the entire cytotoxicity range (Table 1).

Based on these results, sub-cytotoxic concentrations were used in the next assays; specifically, the concentrations values used in the determination of anti-inflammatory response were IC_50_, ¾ IC_50_, ½ IC_50_, and ¼ IC_50_.

### 2.5. Nitric Oxide Production

Having in mind that the ligand and complex are anti-inflammatory compounds, both must show anti-inflammatory properties. Nevertheless, it has been reported that the formation of the anti-inflammatory complex may have some additional advantages over the ligand. Thus, for example, the Zn-aceclofenac complex, reduces the formation of stomach ulcers in comparison to aceclofenac, probably by masking the free carboxylic group of the aceclofenac in the coordination complex. Both Zn-complex and aceclofenac showed abilities to reduce inflammatory edema in the paws of rats challenged by carrageenan, but the Zn-aceclofenac complex induced fewer ulcers in comparison with aceclofenac alone [48].

Several research groups have development different studies to develop new anti-inflammatory complex compounds, thus have been reported Co(II) complexes of deprotonated mefenamic acid, showed scavenger properties from DPPH (2,2-diphenylpicrylhydrazyl). Furthermore, Co(II) complexes of naproxen [49], tolfenamic acid [50], and Mn (II) complexes of tolfenamic, have shown anti-inflammatory properties [51].

In the inflammatory response process, nitric oxide, NO, is released as an intermediate or second messenger. The enzymatic production of NO is cell type specific, with cytokine-driven inducible NOS (iNOS) noted initially for the burst of higher levels as part of the inflammatory activation process. RAW 264.7 murine macrophage cells produce the highest release of NO during the inflammatory response, being an especially indicated model in anti-inflammatory compounds screening studies. As the nitrites concentration is proportional to the NO release, in this study the anti-inflammatory potential of the compounds assayed was analysed by measuring of nitrites in cell culture medium. Firstly, macrophages 264.7 were activated with LPS for 24 h. After this stimulation period cells were incubated with compound **1**, **2**, **3**, and **4**, for 24 h. The Griess reaction was used to determine the nitrite concentration in all the samples as described in Section 3.

**Figure 8:**
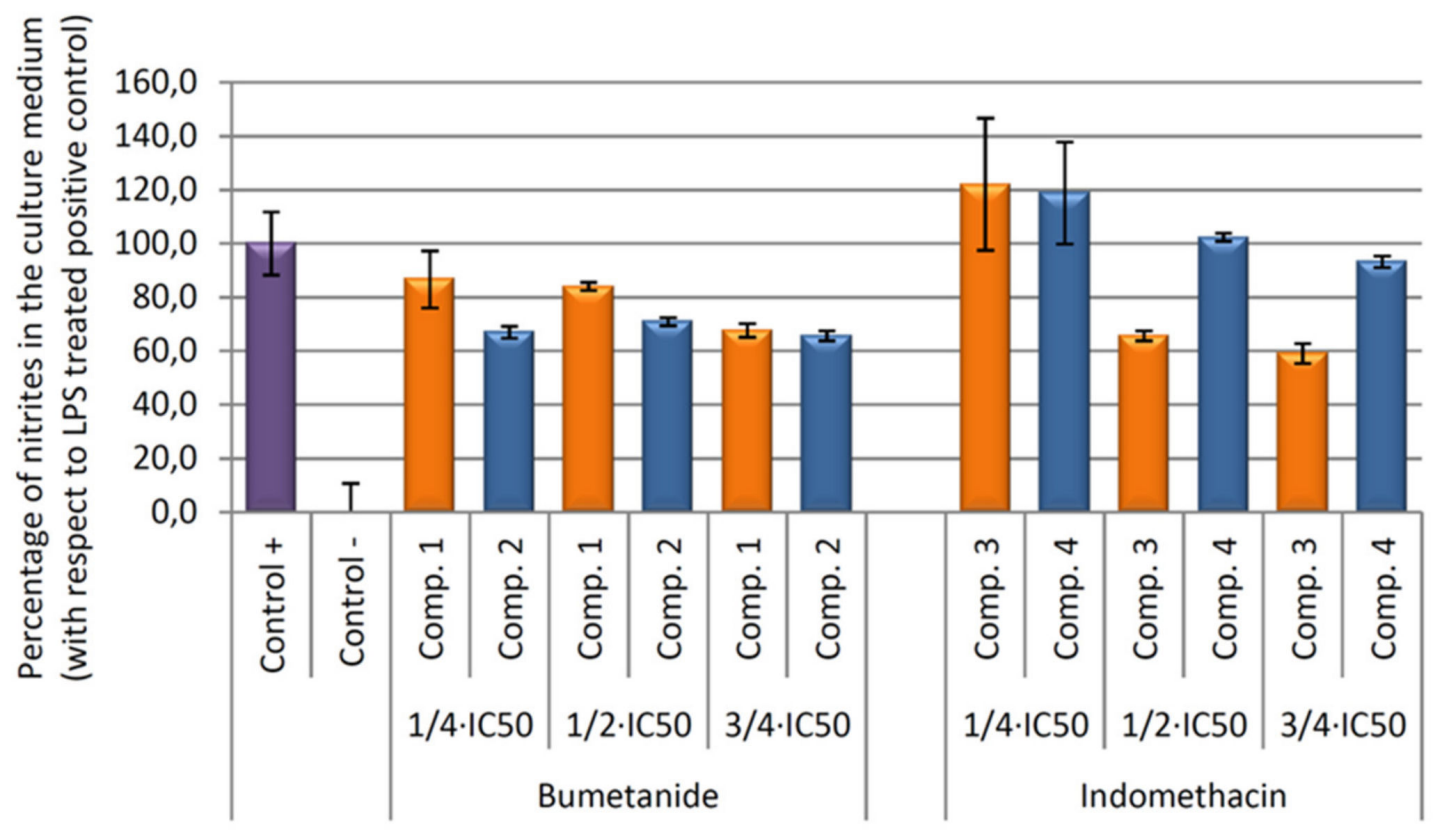
Effect of compounds **1**, **2**, **3**, and **4** on the release of nitrites assay in RAW 264.7 macrophage murine cells. After activation of the inflammatory process by incubation with LPS for 24 h, the compounds were incubated for 24 h at concentrations de ¾ × IC_50_, ½ × IC_50_, ¼ × IC_50_. The data represent the mean ± S.D. of at least two independent experiments performed in triplicate.

Our results showed that all compounds tested produce an inhibition of NO release, being higher in compound **3** versus their vehicle (Figure 8). In depth, after 24 h of incubation, the higher anti-inflammatory effect was produced by the compound **3**, indomethacin-Co complex, with a 40.92% of NO inhibition with respect to the positive control (only LPS treated control cells). This percentage was six times higher than the inhibition achieved only by the indomethacin ligand treatment (compound 4, 6.78% of NO inhibition).

In contrast, bumetanide and bumetanide-cobalt complex showed similar depressed NO levels at 54 μg/mL (¾ × IC_50_ concentration) and non-significant differences were found. At longer incubation times (48 and 72 h), all compounds produced the inhibition of the inflammatory processes, although not significant differences were found between cobalt compounds, compounds **1** and **3**, and its ligand, compounds **2** and **4** (data not show).

**Figure 9:**
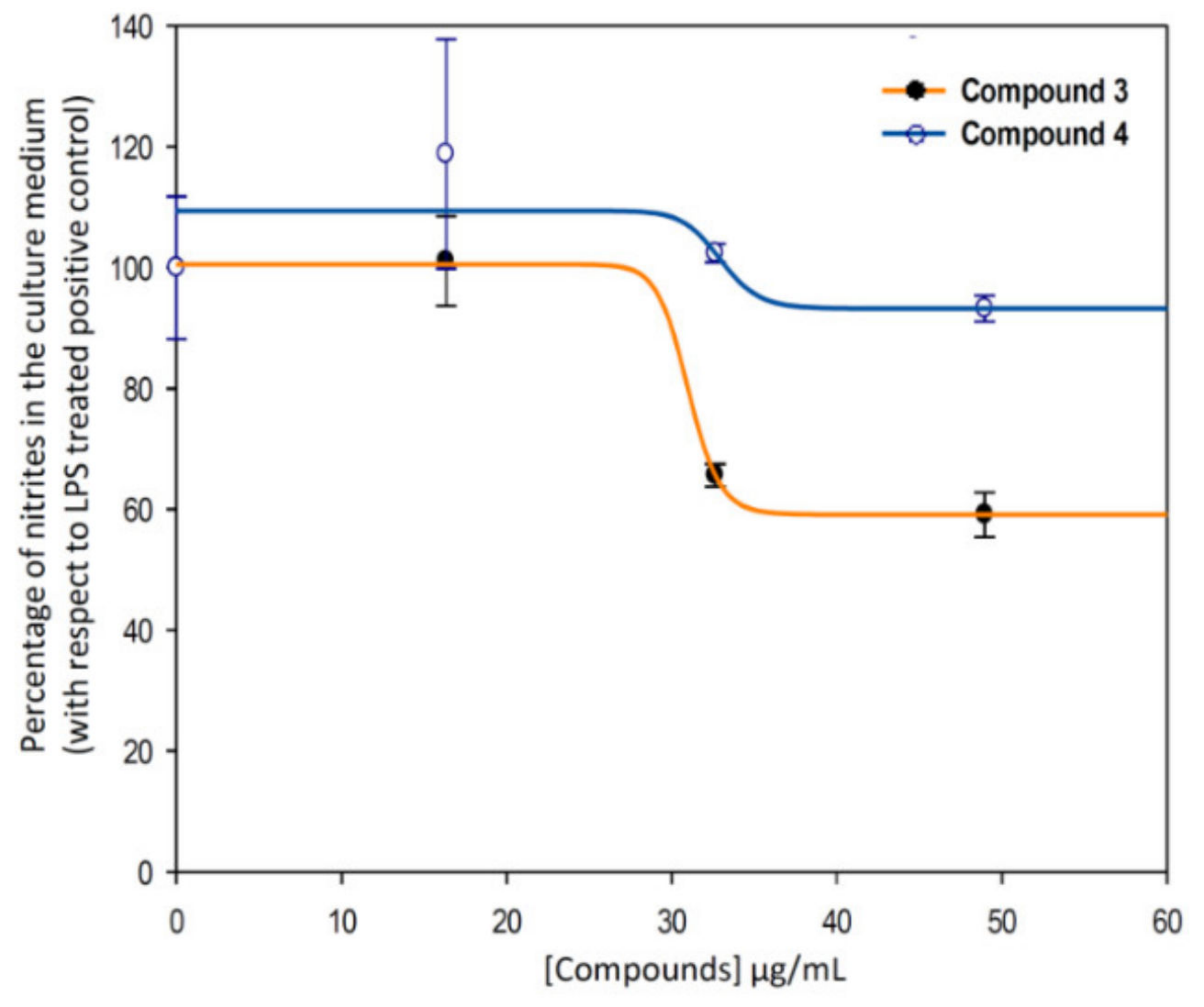
Sigmoidal curves of the effect of the compounds **3** (blue) and **4** (ind, orange) on the release of nitrites in RAW 264.7 macrophage murine cells. Clearly, the curves corresponding to metal-complex compound (**3**, EC50_NO_ = 28.76 ± 4.23 μg/mL) are below to the ligand one (**4**, EC50_NO_ = 35.92 ± 0.97 μg/mL). The data represent the mean ± S.D. of at least two independent experiments performed in triplicate.

Therefore, the anti-inflammatory effect study was deeply performed for compound **3**, and we calculated the effective doses at 50% of NO inhibition (EC50_NO_) after 24 h of treatment of compounds **3** (indomethacin-Co complex), and **4** (indomethacin ligand) (Figure 9). Our data showed that the EC50_NO_ of compound **3** was less than the one found for compound **4**. Concretely, the EC50_NO_ for compound **3** was 28.76 ± 4.23 μg/mL, and the EC50_NO_ for the compound **4** was 35.92 ± 0.97 μg/mL.

### 2.6. Cell Cycle Arrest and Distribution

Flow cytometry is generally used to measure DNA ploidy, as well as alterations in cell-cycle profiles. Differentiation processes increase the cell population in G0/G1 phase where these cells expressed its physiological characteristics. The information obtained by this technique includes the visualization of cell subpopulations with different DNA contents. For each nucleus subpopulation identified, the parameters of population size, fractions of nuclei in each phase of the cell cycle and computation of DNA ratios can be discerned.

**Figure 10:**
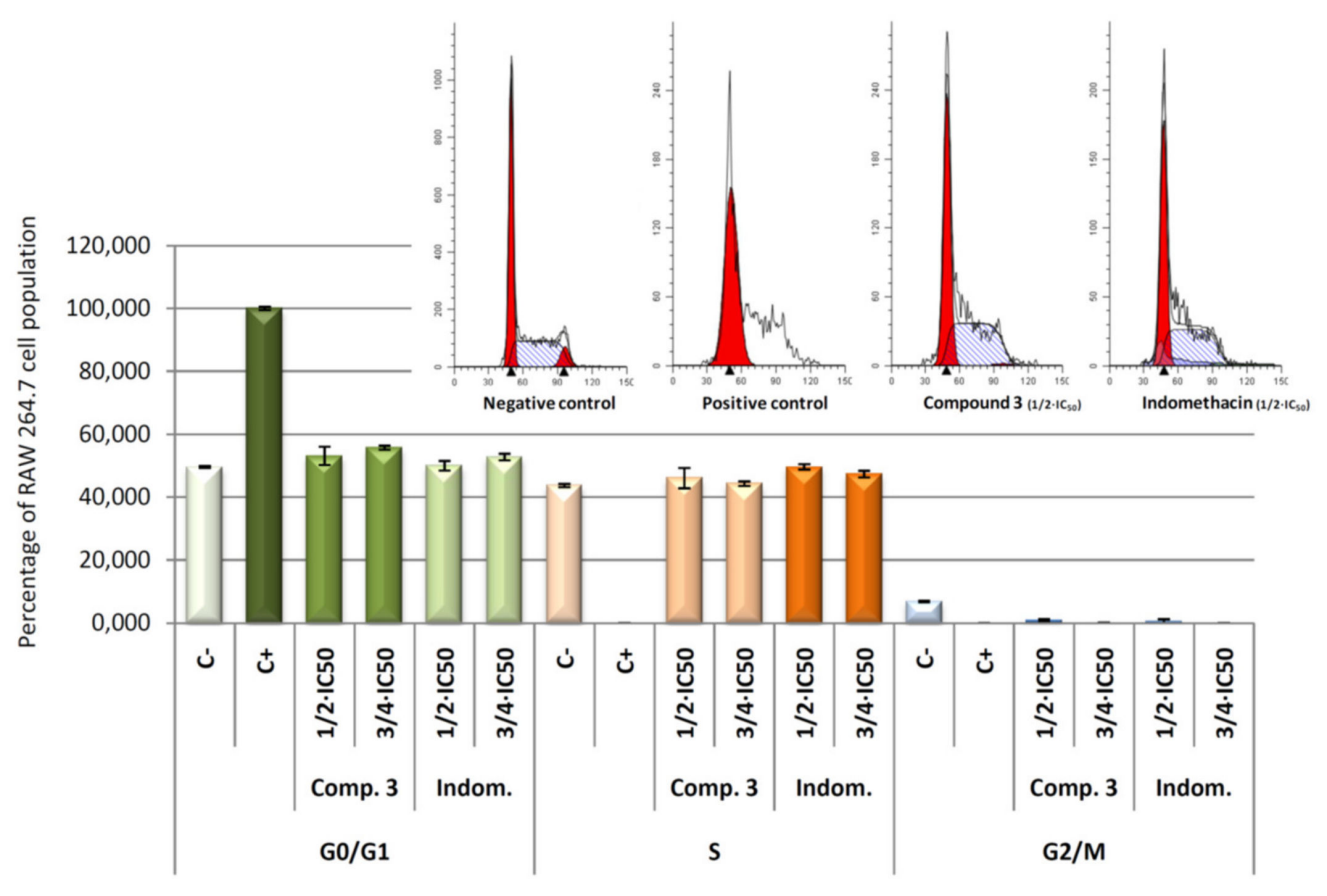
Percentage of RAW 264.7 macrophage murine cells in the different phases of cellular cycle, G0/G1, S and G2/M. Cells were treated with LPS for 24 h, to induced inflammatory process, after that cells were treated with compound **1**, **2**, **3**, and **4** for 24 h at its ½ × IC_50_ and ¾ × IC_50_ concentrations. In addition, results obtained for negative control (untreated cells) and positive control (cells only treated with LPS) are showed. Data represent the mean ± S.D. of at least two independent experiments performed in triplicate.

Concretely, the distribution of cells in each of cell cycle phases was analysed by flow cytometry with propidium iodide (IP) stained (Figure 10). DNA histogram analysis revealed that the four compounds were able to revert the cell cycle arrest induced by LPS. The arrest of cell cycle in LPS-induced RAW 264.7 cells produced a 100% of detention in G0/G1 phase (positive control). After 24 h of treatment with the products **1**, **2**, **3**, and **4**, we observed a decrease of the number of cells in the G0/G1 phase, with the consequent increase of the number of cells in the S phase. Changes in G2/M phase were not significant. This recovery of cell cycle with respect to control cells (only LPS treated) could be a consequence of the anti-inflammatory effects produced by the compounds tested, which lead an increase in cell division. These results showed not significant differences between the products assayed at the concentrations used, indicating that all products assayed present anti-inflammatory effects at the concentrations assayed.

### 2.7. Stoichiometric Mixtures

To determine if the synthetized coordination compounds **1** and **3**, were more effective than the stoichiometric mixture of metal and ligands in the same proportions, we analyzed the liberation of nitric oxide (NO) in response to the following mixture: **M1** (bumetanide and Cobalt), **M2** (indomethacin and cobalt). For these experiments we used cobalt(II) nitrate hexahydrate in a 1:1 stoichiometry with the corresponding ligands. After inflammation induction in murine macrophage/monocyte RAW 264.7 cells with LPS, as we described in Section 3, concentrations of free nitrites in cell medium were determined after 24 h of treatment with increased concentrations of the mixtures (in the same range used for the test of the complex compounds assayed) 17.5, 35.0, and 52.5 μg/mL.

**Figure 11:**
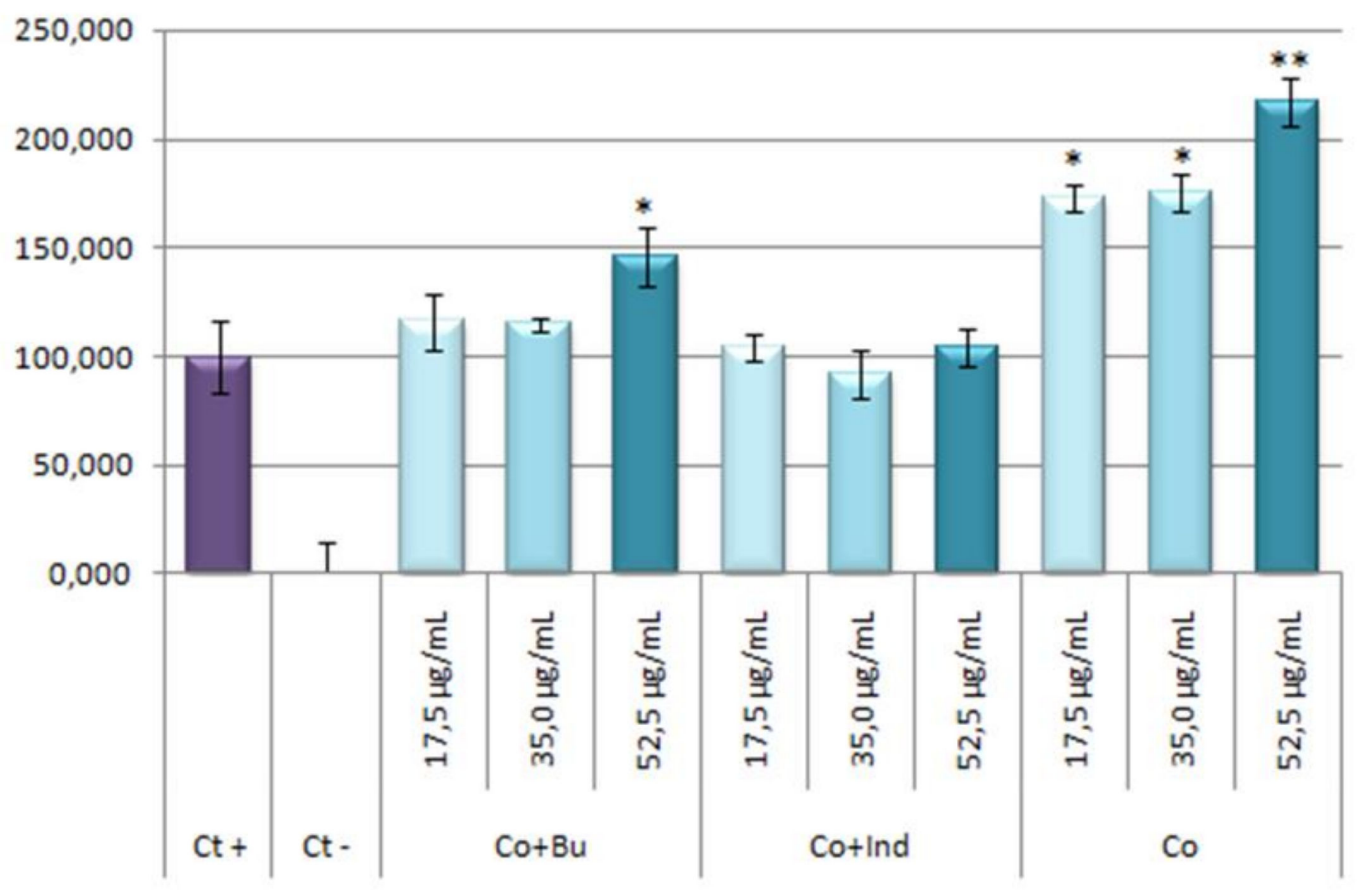
Effect of Co and stoichiometric Co-mixtures with bum and ind on the release of nitrites in LPS RAW 264.7 activated macrophage murine cells. The stoichiometric mixtures were incubated for 24 h at concentrations of 17.5, 35.0, 52.5 μg/mL. The data represent the mean ± S.D. of at least two independent experiments performed in triplicate. Key: *p* < 0.1 (*), *p* ≤ 0.01 (**), respect to LPS treated positive control cells.

Results obtained showed that the stoichiometric mixtures had not any effect on NO production inhibition as response to inflammatory process induced by LPS (Figure 11).

The percentage of NO production in respond to stoichiometric mixtures or cobalt are given in Table 2. Curiously, stoichiometric mixture of cobalt and bumetanide seems to increase the NO production (around a 45% greater than positive control) at the highest concentration, which could be due to a possible pro-inflammatory effect. This surprising response is even more acute in the cobalt treatments reaching values near to 72% higher than positive control at 17.5 and 35.0 μg/mL concentrations, and up to 117% higher than positive control at 52.5 μg/mL concentration. These results showed that the treatment with Co alone resulted be pro-inflammatory. This asseveration has been supported by bibliography; in this way, the pro-inflammatory effect of the cobalt had been observed since the mid-20th century [52,53].

Furthermore, Co^+2^ and cobalt nanoparticles (CoNPs) have showed to induce inflammatory process with increase of reactive oxygen species (ROS) and inflammatory cytokines in Balb/3T3 cells, as TNFα, IL-1β, and IL-6 [54]. The recruit of inflammatory cells have been described in respond to cobalt ions, mediated by TLR4 receptor, increasing the secretions of inflammatory chemokines (IL-8 and CCL2) [55]. These pro-inflammatory effects has been also described in metal-on-metal hip replacements, manufactured from a cobalt-chrome alloy [56]. With respect to our results in the mixtures of the Co and bumetanide or indomethacin, probably these anti-inflammatory compounds counteracted the pro-inflammatory effect of the cobalt, whereas in the treatment with the complexes, the Co ions are completely kidnap by then, avoiding its inflammatory effect.

### 2.8. Stability of Compounds in Solution

Crystals of complexes **1** and **3** were dissolved deuterated dimethylsulfoxide and proved to be stable over several days without any sign of decomposition. The DMSO-*d_6_* ^1^H NMR spectra at 23 °C for both complexes exhibit a number of resonances consistent with paramagnetic species having resonances shifted over a 100 ppm range. In general, the in-plane hydrogens on the chelate exhibit relatively large deviations from their diamagnetic reference values. In **1** for instance, signals located at 88.8 (W1/2 = 382 Hz) and 64.7 ppm (W1/2 = 208 Hz) were observed (Figure S1), whereas in **3**, signals at 88.4 (W1/2 = 930 Hz) and 64.8 ppm (W1/2 = 446 Hz) could be detected (Figure S3). The observation of these signals is uniquivocal proof that the complexes remain in solution. Interestingly, the ^1^H NMR spectrum of complex **3** showed signals associated with diamagnetic ethanol (dH 1.06, 3.46 and 4.32 ppm), which is probably due to their exchange with DMSO solvent.

## 3. Materials and Methods

### 3.1. Materials and Physical Measurements

All reagents were obtained from commercial Sigma-Aldrich source (Spain) and used as received. Elemental (C, H, and N) analyses were performed on a Leco CHNS-932 microanalyzer (Elemental microanalysis Ltd., Okehampton, UK). IR spectra of powdered samples were recorded in the 400–4000 cm^−1^ region on a Nicolet 6700 FTIR spectrophotometer using KBr pellets.

### 3.2. Synthesis of [Co(bum)_2_(H_2_O)_2_](H_2_O)_2_ (1)

A solution containing cobalt(II) acetate tetrahydrate (0.0123) was mixed with other of bumetanide (**2**, 0.0364 g) using the minimum volume of absolute ethanol as solvent, stirring and heating at 70 °C for 20 min. After evaporation at room temperature for 24 h, purple crystals suitable for XRD characterization were obtained by vacuum filtration. Yield: 43%, based on Co. Anal. calcd C_34_H_46_N_4_O_14_S_2_Co: C 47.61, H 5.40, N 6.53. Found: C 47.32, H 5.11, N 7.03.

### 3.3. Synthesis of [Co(ind)_2_(EtOH)_2_] (3)

A solution containing cobalt(II) acetate tetrahydrate (0.0123) was mixed with other of indomethacin (**4**, 0.0359 g) using the minimum volume of ethanol absolute as solvent, stirring and heating at 70 °C during 20 min. After evaporation at room temperature for 48 h, pink crystals suitable for XRD characterization were obtained by vacuum filtration. Yield: 35%, based on Co. Anal. calcd C_42_H_42_N_2_O_10_Cl_2_Co: C 58.34, H 4.90, N 3.24. Found: C 58.24, H 4.58, N 3.42.

### 3.4. Crystallographic Refinement and Structure Solution

Prismatic crystals for **1** and **3** were mounted on a glass fiber and used for data collection on a Bruker D8 Venture with a photon detector equipped with graphite monochromated *MoKα* radiation (λ = 0.71073 Å). The data reduction was performed with the APEX2 [57] software and corrected for absorption using SADABS [58]. Crystal structures were solved by direct methods using the SIR97 program [59] and refined by full-matrix least-squares on *F^2^* including all reflections using anisotropic displacement parameters [60]. The OLEX2 software was used as a graphical interface [61]. Generally, anisotropic temperature factors were assigned to all atoms except for hydrogen atoms, which are riding their parent atoms with an isotropic temperature factor arbitrarily chosen as 1.2 times that of the respective parent. In **1**, only one H-atom of the water molecule was visible from Fourier maps meanwhile the other hydrogen atom was modelled as a positional disorder in two alternative positions (0.56:0.44 ratio). Final R(*F*), *w*R(*F*^2^) and goodness of fit agreement factors, details on the data collection and analysis can be found in Table 3. CCDC numbers are 1836761 and 1836762 for **1** and **3**, respectively. These data can be obtained free of charge from The Cambridge Crystallographic Data Centre via [62].

### 3.5. Experiments in the Nematode Caenorhabditis Elegans

*C. elegans* strain Bristol N2 (wild-type), as well as the *Escherichia coli* OP50 strain were obtained from the Caenorhabditis Genetics Center, University of Minnesota (Minneapolis–St. Paul, MN, USA). Worms were maintained at 20 °C on nematode growth medium (NGM) agar plates carrying a lawn of *E. coli* OP50. Synchronization of worm cultures was achieved by hypochlorite treatment of gravid hermaphrodites. Two different analyses were performed on nematodes. First, a lethality assay [63] was performed to determine the death rate derived from acute toxicity in a concentration-response curve basis. A total of 10 ± 1 young adults were transferred into 24-well microplates, which contained concentrations of compound **1** or compound **3** (ranging from 0 to 200 µM) and a negative control. The exposure was carried out at 20 °C during 24 h in the absence of food. Then, the number of live and dead worms was counted through visual inspection using a dissecting microscope. Death was assumed when there was no movement during an observation period of 30 s. The second assay consisted on the measurement of glucose content in the worms after four days of exposure to the experimental compounds or to a negative control. After experimental exposure, 20 worms were picked into a vial containing 200µL of ice-PBS buffer and centrifuged at 600× *g* to remove rest of food and compounds. After removing the supernatant, worms were added with 100 µL of ice-PBS and homogenized by a glass homogenizer. The resulting homogenate was used to measure glucose content by a Spinreact kit (Spinreact: Girona, Spain). Glucose content was normalized by protein content, which was measured using Pierce^®^ BCA Protein Assay (Thermo Scientific: Rockford, IL, USA). Glucose and protein content were measured in 96-well microplates in a Synergy Neo2 multiplate reader (Biotek Instruments, Winooski, VT, USA). Lethality and glucose content experiments were performed in triplicate. Statistical differences between compounds exposure with respect to the control were tested by Student’s *t* test. In all analyses, significant differences were established at *p* < 0.05. Statistical analysis was performed with SPSS 24.0 for Windows (IBM: Chicago, IL, USA).

### 3.6. Drugs

Compounds **1**, **2**, **3**, and **4** were dissolved in DMSO at 5 mg/mL and stored at 4 °C. Before the treatment the stock solutions were diluted in cell-culture medium to the adequate concentrations for each experiment. In the *C. elegans* experiments drugs were used at a range concentration from 0 to 200 µM as indicated. For nitrite concentration, ¾ × IC_50_, ½ × IC_50_, ¼ × IC_50_ concentrations were used (IC_50_, concentration causing 50% reduction growth). For cell cycle analysis, ¾ × IC_50_ and ½ × IC_50_ were used. All experiments were measured and compared to control untreated cells after 24, 48, or 72 h of treatment.

### 3.7. Cell Culture

A monocyte-lineage cell line was used, the murine macrophage-like RAW 264.7 cell line (ECACC no; ATCC no) was cultured in DMEM medium supplemented with 2 mM glutamine, 10% heat-inactivated FCS, 10,000 units/mL of penicillin, and 10 mg/mL of streptomycin, being incubated at 37 °C in an atmosphere of 5% CO_2_ and 95% humidity. Cells were grown to 80–90% of confluence in sterile cell culture flasks. Sub-confluent monolayer cells were used in all experiments. The cell line was provided by cell bank of the University of Granada, Spain.

### 3.8. Cell Viability Assay

The effect of each treatment with compounds **1**, **2**, **3**, or **4** in RAW 264.7 murine macrophage cells was measured using the MTT (3-(4,5-dimethylthiazol-2-yl)-2,5-diphenyltetrazolium bromide) proliferation assay (Sigma, MO, USA), which is based on the ability of live cells to cleave the tetrazolium ring, thus producing formazan, which absorbs at 570 nm [64].

Cell viability was determined by measuring the absorbance of MTT dye staining of living cells. For this assay, 6 × 10^3^ RAW 264.7 cells were grown on a 96-well plate and incubated with compounds **1**, **2**, **3**, and **4** respectively, at different concentrations (0–100 µg/mL). Lately, after 72 h of incubation, 100 µL of MTT solution (0.5 mg/mL) in 50% of PBS 50% of medium was added to each well. After 1.5 h of incubation formazan was re-suspended in 100 µL of DMSO. Relative cell viability, with respect to untreated control cells, was measured by absorbance at 570 nm on an ELISA plate reader (Tecan Sunrise MR20–301, TECAN, Austria).

### 3.9. Determination of Nitrite Concentration

Nitrite concentration was used as indicator of NO production. NO determination was based on Griess reaction [65].

Cells were plated at 6 × 10^4^ cells/well in 24-well cell culture plates and supplemented with 10 µg/mL of LPS. After 24 h of plated, cells were incubated for 24 h with compounds **1**, **2**, **3**, and **4** at ¾ × IC_50_, ½ × IC_50_, ¼ × IC_50_ concentrations. The supernatants were collected to determine their nitrite concentration and/or stored at –80 °C for further use.

Griess reaction was performed taking 150 µL of supernatant test samples or sodium nitrite standard (0–120 µM) and mixed with 25 µL of Griess reagent A (0.1% N-N-(1-naphthyl)-ethylenediamine dihydrochloride) and 25 µL of Griess reagent B (1% sulfanilamide in 5% of phosphoric acid), in a 96-well plate. After 15 min of incubation at room temperature, the absorbance was measure at 540 nm in an ELISA plate reader (Tecan Sunrise MR20-301, TECAN, Austria). The absorbance was referred to nitrite standard curve to determine the concentration of nitrite in the supernatant of each experimental sample. The percentage of NO production was determined, assigning 100% at the increase between negative control (untreated cells) and positive control (cells only treated with 10 µg/mL of LPS).

### 3.10. Cell Cycle Analysis

Flow cytometry is a quick novel method to determine efficiently and reproducibly the relative DNA content, ploidy as well as to measure alterations in cell cycle profiles. DNA content is directly proportional to the PI fluorescence, which allows to determine the percentage of cell in each cell cycle phase. In this way, we could visualize cellular subpopulations, with different DNA contents. Changes in DNA levels are characteristic of cell cycle arrest and cell differentiation.

The number of cells in each stage of the cell cycle was estimated by fluorescence-associated cell sorting (FACS) at 488 nm in an Epics XL flow cytometer (Coulter Corporation, Hialeah, FL, USA). For this assay 12 × 10^4^ RAW 264.7 murine macrophage/monocyte cells stimulated with LPS were plated in 24-well plates with 1.5 mL of medium and incubated with the compounds under study for 24 h, at ¾ × IC_50_ and ½ × IC_50_ concentrations, as we described above. Several controls were analyzed in a parallel way; a positive control was performed with cells treated only with LPS stimulation, and a negative control where RAW 264.7 cells were exposed to the compounds under study without lipopolysacharide stimulation. After treatment, cells were washed twice with PBS and harvested by tripsinization, then were re-suspended in TBS 1X (10Mm Tris, 150Mm NaCl) and subsequently added Vindelov Buffer (100 mM Tris, 100 MmNaCl, 10 mg/mL Rnasa, 1 mg/mL PI, pH 8). The samples were allowed to stand for 15 min on ice. Immediately before FACS analysis, cells were stained with 20 µL of 1 mg/mL PI solution. Data were analyzed to determine the percentage of cells in each cell cycle phase (G0/G1, S, and G2/M).

### 3.11. Statistics

Statistical and non-lineal regression analyses were performed with the SigmaPlot 12.5 software. All quantitative data were summarized as the means ± standard deviation (SD). All data shown here were representative of at least two independent experiments performed in triplicate.

## 4. Conclusions

Two novel cobalt coordination compounds using bumetanide and indomethacin therapeutic agents have been synthesized. These compounds present mononuclear structures and show slow relaxation of the magnetization. Regarding the anti-inflammatory assays, were performed under sub-citotoxic concentrations of the compounds in this study (**1**, **2**, **3**, and **4**) to ensure that the possible anti-inflammatory effect was exclusively due to their anti-inflammatory properties and not to their cytotoxic effects. For this reason ¾ IC_50_, ½ IC_50_, and ¼ IC_50_ concentrations were used in the further assays. All products assayed present anti-inflammatory effects at the concentrations used, being able to revert the 100% cell cycle arrest in the G0/G1 phase induced by LPS in RAW 264.7 murine monocyte/macrophage cells, however, no significant differences were found in the cell cycle effects for the different products assayed. Moreover, our results showed that indomethacin-Co complex, was six times more effective than its ligand (indomehtacine) in the inhibition of NO production, whereas non-significant differences in the NO inhibition were found between bumetanide-Co complex and bumetanide. The EC50 _NO_, for the compound **3** was calculated being 28.76 ± 4.23 μg/mL, after 24 h of treatment. Concerning antidiabetic properties, results suggest that compounds **1** and **3** are endowed with antidiabetic properties as demonstrated by the lower glucose content found in *C. elegans* when compared with non-treated nematodes. For future in vivo studies, it remains to be analyzed whether the new complexes modify in any way the side effects shown by indomethacin and bumetanide at the gastrointestinal level. In conclusion, our results reveal that the synthesis of metal complexes with bioactive ligands is a new and promising strategy to find new compounds with high and enhanced biochemical properties.

## Figures and Tables

**Table 1:** Growth-inhibitory effects of compounds **1**, **2**, **3**, and **4** on RAW 264.7 monocyte/macrophage murine cells.

Compound	IC_20_	IC_50_	IC_80_
**1**	54.04 ± 6.31	72.10 ± 6.63	103.63 ± 10.4
**2**	55.83 ± 6.98	71.74 ± 9.64	97.16 ± 0.43
**3**	54.54 ± 3.61	65.27 ± 1.41	78.49 ± 3.82
**4**	58.37 ± 6.09	72.55 ± 4.12	93.48 ± 3.04

**Table 2:** Percentage of release of nitrites in LPS RAW 264.7 activated cells in respond to stoichiometric mixtures and Co.

Mixture	17.5 μg/mL	35.0 μg/mL	52.5 μg/mL
**Co+Bu**	116.39 ± 13.01	114.75 ± 3.28	145.90 ± 13.01
**Co+Ind**	104.10 ± 6.19	91.80 ± 10.62	104.10 ± 8.40
**Co**	175.41 ± 6.35	175.41 ± 8.52	217.21 ± 10.91

**Table 3:** Crystallographic data and structure refinement details of all compounds.

Compound	1	3
Chem. form.	C_34_H_46_N_4_O_14_S_2_Co	C_42_H_42_Cl_2_N_2_O_10_Co
Form. weight	857.80	864.60
Cryst. system	Monoclinic	Monoclinic
Space group	*P*2/*c*	*C*2/c
*a* (Å)	20.470(3)	30.137(4)
*b* (Å)	5.267(2)	5.388(2)
*c* (Å)	18.294(3)	23.908(3)
*α* (°)	90	90
*β* (°)	96.252(3)	91.339(4)
*γ* (°)	90	90
V (Å^3^)	1960.8(9)	3881.0(17)
Z	2	4
GOF ^a^	1.077	1.034
Rint	0.1130	0.1023
R_1_^b^ / wR^2 c^ [I > 2σ(I)]	0.0616 / 0.1269	0.0390 / 0.0662
R_1_^b^ / wR^2 c^ (all data)	0.0971 / 0.1420	0.0662 / 0.0740

[a] S = [∑w(F_0_^2^ – F_c_^2^)^2^ / (N_obs_ – N_param_)]^1/2^ [b] R_1_ = ∑||F_0_|–|F_c_|| / ∑|F_0_| [c] wR_2_ = [∑w(F_0_^2^ – F_c_^2^)^2^ / ∑wF_0_^2^]^1/2^; w = 1/[σ^2^(F_0_^2^) + (aP)^2^ + bP] where *p* = (max(F_0_^2^,0) + 2Fc^2^)/3

## Supplementary Materials

Supplementary materials can be found at https://www.mdpi.com/1422-0067/21/9/3146/s1. Table S1. Bond distances for [Co(bum)_2_(H_2_O)_2_](H_2_O)_2_ (1). Table S2. Hydrogen bonds for compound 1 [Å and °]. Table S3. Bond distances and hydrogen bonds for [Co(ind)_2_(EtOH)_2_] (3). Table S4. CShMs for the CoO_6_ coordination environment of compounds 1 and 3. The lowest SHAPE values for each ion are shown highlighted in grey, indicating best fits. Figure S1. ^1^H NMR (300 MHz) spectrum of complex [Co(bum)_2_(H_2_O)_2_](H_2_O)_2_ in DMSO-*d*_6_ at 23 °C. Figure S2. ^1^H NMR (300 MHz) spectrum of complex [Co(ind)_2_(EtOH)_2_] in DMSO-*d*_6_ at 23 °C. Figure S3. Chains constructed of mononuclear entities by hydrogen bonds in compound 1. Figure S4. Chains constructed of mononuclear entities by hydrogen bonds in compound 3.

## Abbreviations

NO	Nitric oxide
MAPK	Mitogen-activated protein kinases
JAK	Janus-activated kinases
PI3K/AKT	Phosphatidylinositol-3-kinase
STAT	Signal transducer activator of transcription
NF-kB	Nuclear factor kappa B
AP-1	Activation proteins 1
HIF-1	Hypoxia inducible factor 1-α
iNOS	Nitric oxide synthase inducible
COX-2	Cyclooxyenase-2
LPS	Bacterial lipopolysaccharide
TNFα	Tumor necrosis factor α
INF- γ	Interferon-γ
NSAID	Nonsteroidal anti-inflammatory drug
COX	Cyclooxygenase enzyme
CCDC	Cambridge Crystallographic Data Centre
QTM	Quantum tunneling of the magnetization
ZFS	Zero-field splitting
SIM	Single ion magnet
DPPH	2,2-Diphenylpicrylhydrazyl
IC50	Total concentration to obtain a 50% growth inhibition
EC50	Dosages that give around 50% of the maximum possible drug effect
IP	Propidium iodide
ROS	Reactive oxygen species

## References

[1] Bastaki S. (2005). Diabetes mellitus and its treatment. Int. J. Diabetes Metab..

[2] Doucette K.A., Hassell K.N., Crans D.C. (2016). Selective speciation improves efficacy and lowers toxicity of platinum anticancer and vanadium antidiabetic drugs. J. Inorg. Biochem..

[3] Joseph J., Janaki G.B., Dharmaraja J. (2016). Metal complexes of 2-aminobenzothiazole derivatives as a versatile system tuning up their structural and biological properties. J. Chem. Pharm. Res..

[4] Tsave O., Yavropoulou M.P., Kafantari M., Gabriel C., Yovos J.G., Salifoglou A. (2018). Comparative assessment of metal-specific adipogenic activity in zinc and vanadium-citrates through associated gene expression. J. Inorg. Biochem..

[5] Tsirulnikova N.V., Podmareva O.N. (2015). Metal Complexes with Ethylenediaminedicarboxylic Acids and Their Derivatives, Promising Pharmacological and Diagnostic Agents. Pharm. Chem. J..

[6] Caballero A.B., Rodriguez-Dieguez A., Quiros M., Salas J.M., Huertas O., Ramirez-Macias I., Olmo F., Marin C., Chaves-Lemaur G., Gutierrez-Sanchez R. (2014). Triazolopyrimidine compounds containing first-row transition metals and their activity against the neglected infectious Chagas disease and leishmaniasis. Eur. J. Med. Chem..

[7] Méndez-Arriaga J.M., Oyarzabal I., Escolano G., Rodríguez-Diégue A., Sánchez-Moreno M., Salas J.M. (2018). In vitro leishmanicidal and trypanocidal evaluation and magnetic properties of 7-amino-1,2,4-triazolo[1 ,5-a]pyrimidine Cu(II) complexes. J. Inorg. Biochem..

[8] Fernández B., Oyarzabal I., Fischer-Fodor E., Macavei S., Sánchez I., Seco J.M., Gómez-Ruiz S., Rodríguez-Diéguez A. (2016). Multifunctional applications of a dysprosium based metal–organic chain with single-ion magnet behavior. CrystEngComm.

[9] Briones D., Fernández B., Calahorro A.J., Fairen-Jimenez D., Sanz R., Martínez F., Orcajo G., San Sebastián E., Seco J.M., Sánchez González C. (2016). Highly Active Anti-Diabetic Metal−Organic Framework. Cryst. Growth Des..

[10] Fernández B., Gómez-Vílchez A., Sánchez-González C., Bayon J., San Sebastián E., Gómez-Ruiz S., López-Chaves C., Aranda P., Llopis J., Rodríguez-Diéguez A. (2016). Novel anti-diabetic and luminescent coordination compounds based on vanadium. New J. Chem..

[11] Jansen J., Karges W., Rink L. (2009). Zinc and diabetes clinical links and molecular mechanisms. J. Nutr. Biochem..

[12] Thompson K.H., Lichter J., Lebel C., Scaife M.C., McNeill J.H., Orvig C. (2009). Vanadium treatment of type 2 diabetes: A view to the future. J. Inorg. Biochem..

[13] Farrell P. (2002). Biomedical uses and applications of inorganic chemistry. An overview. Coord. Chem. Rev..

[14] Pollack R.M., Donath M.Y., LeRoith D., Leibowitz G. (2016). Anti-inflammatory Agents in the Treatment of Diabetes and Its Vascular Complications. Diabetes Care.

[15] Lucas S. (2016). The Pharmacology of Indomethacin. Headache.

[16] Castellano A.E., Micieli G., Bellantonio P., Buzzi M.G., Marcheselli S., Pompeo F., Rossi F., Nappi G. (1998). Indomethacin increases the effect of isosorbide dinitrate on cerebral hemodynamic in migraine patients: Pathogenetic and therapeutic implications. Cephalalgia.

[17] Yeh K.C. (1985). Pharmacokinetic Overview of Indomethacin and Sustained-Release Indomethacin. Am. J. Med..

[18] Topper J.N., Wasserman S.M., Anderson K.R., Cai J., Falb D., Gimbrone M.A. (1997). Expression of the bumetanide-sensitive Na-K-Cl cotransporter BSC2 is differentially regulated by fluid mechanical and inflammatory cytokine stimuli in vascular endothelium. J. Clin. Investig..

[19] Lemonnier E., Villeneuve N., Sonie S., Serret S., Rosier A., Roue M., Brosset P., Viellard M., Bernoux D., Rondeau S. (2017). Effects of bumetanide on neurobehavioral function in children and adolescents with autism spectrum disorders. Transl. Psychiatry.

[20] Reddy M.M., Quinton P.M. (1999). Bumetanide blocks CFTR GCl in the native sweat duct. Am. J. Physiol..

[21] Ong W., Cheung E.Y., Schultz K.A., Smith C., Bourassa J., Hickey M.B. (2012). Sodium and potassium salts of bumetanide trihydrate: Impact of counterion on structure, aqueous solubility and dehydration kinetics. CrystEngComm.

[22] Morgan Y.R., Turner P., Kennedy B.J., Hambley T.W., Lay P.A., Biffin J.R., Regtop H.L., Warwick B. (2001). Preparation and characterization of dinuclear copper-indomethacin anti-inflammatory drugs. Inorg. Chim. Acta.

[23] Galani A., Kovala-Demertzi D., Kourkoumelis N., Koutsodimou A., Dokorou V., Ciunik Z., Russo U., Demertzis M.A. (2004). Organotin adducts of indomethacin: Synthesis, crystal structures and spectral characterization of the first organotin complexes of indomethacin. Polyhedron.

[24] Zhou Q., Hambley T.W., Kennedy B.J., Lay P.A., Turner P., Warwick B., Biffin J.R., Regtop H.L. (2000). Syntheses and Characterization of Anti-inflammatory Dinuclear and Mononuclear Zinc Indomethacin Complexes. Crystal Structures of [Zn2(Indomethacin)4(L)2] (L = N,N-Dimethylacetamide, Pyridine, 1-Methyl-2-pyrrolidinone) and [Zn(Indomethacin)2(L1)2] (L1 = Ethanol, Methanol). Inorg. Chem..

[25] Iwahara N., Chibotaru L.F. (2016). New mechanism of kinetic exchange interaction induced by strong magnetic anisotropy. Sci. Rep..

[26] Chakarawet K., Bunting P.C., Long J.R. (2018). Large Anisotropy Barrier in a Tetranuclear Single-Molecule Magnet Featuring Low-Coordinate Cobalt Centers. J. Am. Chem. Soc..

[27] Campbell V.E., Tonelli M., Cimatti I., Moussy J.-B., Tortech L., Dappe Y.J., Riviere E., Guillot R., Delprat S., Mattana R. (2016). Engineering the magnetic coupling and anisotropy at the molecule-magnetic surface interface in molecular spintronic devices. Nat. Commun..

[28] Lloret F., Julve M., Cano J., Ruiz-Garcia R., Pardo E. (2008). Magnetic properties of six-coordinated high-spin cobalt (II) complexes: Theoretical background and its application. Inorg. Chim. Acta.

[29] Vallejo J., Castro I., Cañadillas-Delgado L., Ruiz-Pérez C., Ferrando-Soria J., Ruiz-García R., Cano J., Lloret F., Julve M. (2010). Ferromagnetic coupling and magnetic anisotropy in oxalato-bridged trinuclear chromium (III)-cobalt (II) complexes with aromatic diimine ligands. Dalton Trans..

[30] Peng Y., Mereacre V., Anson C.E., Zhang Y., Bodenstein T., Fink K., Powell A.K. (2017). Field-Induced Co(II) Single-Ion Magnets with mer-Directing Ligands but Ambiguous Coordination Geometry. Inorg. Chem..

[31] Rodríguez-Diéguez A., Pérez-Yáñez S., Ruiz-Rubio L., Seco J.M., Cepeda J. (2017). From isolated to 2D coordination polymers based on 6-aminonicotinate and 3d-metal ions: Towards field-induced single-ion-magnets. CrystEngComm.

[32] Wang Y.-L., Chen L., Liu C.-M., Du Z.-Y., Chen L.-L., Liu Q.-Y. (2016). 3D chiral and 2D achiral cobalt(II) compounds constructed from a 4-(benzimidazole-1-yl)benzoic ligand exhibiting field-induced single-ion-magnet-type slow magnetic relaxation. Dalton Trans..

[33] Frost J.M., Harriman K.L.M., Murugesu M. (2016). The Rise of 3-d Single-Ion Magnets in Molecular Magnetism: Towards Materials from Molecules. Chem. Sci..

[34] Craig G.A., Murrie M. (2015). 3d Single-Ion Magnets. Chem. Soc. Rev..

[35] Liu X., Sun L., Zhou H., Cen P., Jin X., Xie G., Chen S., Hu Q. (2015). Single-Ion-Magnet Behavior in a Two-Dimensional Coordination Polymer Constructed from CoII Nodes and a Pyridylhydrazone Derivative. Inorg. Chem..

[36] Vallejo J., Castro I., Ruiz-García R., Cano J., Julve M., Lloret F., De Munno G., Wernsdorfer W., Pardo E. (2012). Field-Induced Slow Magnetic Relaxation in a Six-Coordinate Mononuclear Cobalt(II) Complex with a Positive Anisotropy. J. Am. Chem. Soc..

[37] Zhu Y.Y., Cui C., Zhang Y.Q., Jia J.H., Guo X., Gao C., Qian K., Jiang S.D., Wang B.W., Wang Z.M. (2013). Zero-Field Slow Magnetic Relaxation from Single Co(Ii) Ion: A Transition Metal Single-Molecule Magnet with High Anisotropy Barrier. Chem. Sci..

[38] Shrivastava K.N. (1983). Theory of spin-lattice relaxation. Phys. Status Solidi B.

[39] García-Valdivia A.A., Seco J.M., Cepeda J., Rodríguez-Diéguez A. (2017). Designing Single-Ion Magnets and Phosphorescent Materials with 1-Methylimidazole-5-carboxylate and Transition-Metal Ions. Inorg. Chem..

[40] Roy S., Oyarzabal I., Vallejo J., Cano J., Colacio E., Bauza A., Frontera A., Kirillov A.M., Drew M.G.B., Das S. (2016). Two Polymorphic Forms of a Six-Coordinate Mononuclear Cobalt(II) Complex with Easy-Plane Anisotropy: Structural Features, Theoretical Calculations, and Field-Induced Slow Relaxation of the Magnetization. Inorg. Chem..

[41] Shi Y.-C., Liao V.H.C., Pan T.M. (2012). Monascin from red mold dioscorea as a novel antidiabetic and antioxidative stress agent in rats and Caenorhabditis elegans. Free Radic. Biol. Med..

[42] Schlotterer A., Kukudov G., Bozorgmehr F., Hutter H., Du X., Oikonomou D., Ibrahim Y., Pfisterer F., Rabbani N., Thornalley P. (2009). C. elegans as model for the study of high glucosemediated life span reduction. Diabetes.

[43] Vasudevan H., McNeill J.H. (2007). Chronic cobalt treatment decreases hyperglycemia in streptozotocin-diabetic rats. Biometals.

[44] Medina-O’Donnell M., Rivas F., Reyes-Zurita F.J., Martinez A., Galisteo-Gonzalez F., Lupianez J.A., Parra A. (2017). Synthesis and in vitro antiproliferative evaluation of PEGylated triterpene acids. Fitoterapia.

[45] Reyes-Zurita F.J., Medina-O’Donnell M., Ferrer-Martin R.M., Rufino-Palomares E.E., Martin-Fonseca S., Rivas F., Martinez A., Garcia-Granados A., Perez-Jimenez A., Garcia-Salguero L. (2016). The oleanolic acid derivative, 3-O-succinyl-28-O-benzyl oleanolate, induces apoptosis in B16-F10 melanoma cells via the mitochondrial apoptotic pathway. RSC Adv..

[46] Fernandez B., Fernandez I., Cepeda J., Medina-O’Donnell M., Rufino-Palornares E.E., Raya-Baron A., Gomez-Ruiz S., Perez-Jimenez A., Lupianez J.A., Reyes-Zurita F.J. (2018). Modulating Anticancer Potential by Modifying the Structural Properties of a Family of Zinc Metal-Organic Chains Based on 4-Nitro-1H-pyrazole. Cryst. Growth Des..

[47] Leung C.H., Lin S., Zhong H.J., Ma D.L. (2015). Metal complexes as potential modulators of inflammatory and autoimmune responses. Chem. Sci..

[48] Kale M.A., Shelke R., Nawale R.B. (2014). Zinc-aceclofenac complex: Synthesis, hydrolysis study and antiinflammatory studies. Antiinflamm. Antiallergy Agents Med. Chem..

[49] Dimiza F., Papadopoulos A.N., Tangoulis V., Psycharis V., Raptopoulou C.P., Kessissoglou D.P., Psomas G. (2012). Biological evaluation of cobalt(II) complexes with non-steroidal anti-inflammatory drug naproxen. J. Inorg. Biochem..

[50] Tsiliou S., Kefala L.A., Perdih F., Turel I., Kessissoglou D.P., Psomas G. (2012). Cobalt(II) complexes with non-steroidal anti-inflammatory drug tolfenamic acid: Structure and biological evaluation. Eur. J. Med. Chem..

[51] Zampakou M., Rizeq N., Tangoulis V., Papadopoulos A.N., Perdih F., Turel I., Psomas G. (2014). Manganese(II) Complexes with the Non-steroidal Anti-Inflammatory Drug Tolfenamic Acid: Structure and Biological Perspectives. Inorg. Chem..

[52] Wintrobe M.M., Grinstein M., Dubash J.J., Humphreys S.R., Ashembrucker H., Worth W. (1947). The Anemia of Infection. 6. The Influence of Cobalt on the Anemia Associated with Inflammation. Blood.

[53] Gubler C.J., Cartwright G.E., Wintrobe M.M. (1950). Influence of Inflammation, Cobalt Administration, Diet and Pyridoxine Deficiency on Iron Absorption. Am. J. Med..

[54] Yan X., Liu Y., Xie T., Liu F. (2018). Alpha-Tocopherol protected against cobalt nanoparticles and cocl(2) induced cytotoxicity and inflammation in Balb/3T3 cells. Immunopharmacol. Immunotoxicol..

[55] Lawrence H., Mawdesley A.E., Holland J.P., Kirby J.A., Deehan D.J., Tyson-Capper A.J. (2016). Targeting Toll-like receptor 4 prevents cobalt-mediated inflammation. Oncotarget.

[56] Samelko L., Landgraeber S., McAllister K., Jacobs J., Hallab N.J. (2016). Cobalt Alloy Implant Debris Induces Inflammation and Bone Loss Primarily through Danger Signaling, Not TLR4 Activation: Implications for DAMP-ening Implant Related Inflammation. PLoS ONE.

[57] (2012). Bruker Apex2.

[58] Krause L., Herbst-Irmer R., Sheldrick G.M., Stalke D. (2015). Comparison of silver and molybdenum microfocus X-ray sources for single-crystal structure determination. J. Appl. Cryst..

[59] Altomare A., Burla M.C., Camilla M., Cascarano G.L., Giacovazzo C., Guagliardi A., Moliterni A.G.G., Polidori G., Spagna R. (1999). SIR97: A new tool for crystal structure determination and refinement. J. Appl. Crystallogr..

[60] Sheldrick G.M. (2015). Crystal structure refinement with SHELXL. Acta Cryst..

[61] Dolomanov O.V., Bourhis L.J., Gildea R.J., Howard J.A.K., Puschmann H. (2009). OLEX2: A Complete Structure Solution, Refinement and Analysis Program. J. Appl. Crystallogr..

[62] The Cambridge Crystallographic Data Centre. www.ccdc.cam.ac.uk/data_request/cif.

[63] Tejeda-Benitez L., Olivero-Verbel J. (2016). Caenorhabditis elegans, a Biological Model for Research in Toxicology. Rev. Environ. Contam. Toxicol..

[64] Mosmann T. (1983). Rapid Colorimetric Assay for Cellular Growth and Survival: Application to Proliferation and Cytotoxicity Assays. J. Immunol. Methods.

[65] Bryan N.S., Grisham M.B. (2007). Methods to Detect Nitric Oxide and its Metabolites in Biological Samples. Free Radicbiol. Med..

