# Supplementary material for: Designing Single-Molecule Magnets as Drugs with Dual Anti-Inflammatory and Anti-Diabetic Effects
Source: Int J Mol Sci. 2020 Apr 29;21(9):3146. doi: 10.3390/ijms21093146 (PMC7246571; doi:10.3390/ijms21093146)
Supplement: Supplementary file 1 [file ijms-21-03146-s001.zip › ijms-785055-supplementary/Supplementary_Files/ESI_Article_IJMS.docx]

Supporting Information for:

Designing Single-Molecule Magnets as Drugs with Dual Anti-Inflammatory and Anti-Diabetic Effects

Arturo Navas, Fatin Jannus, Belén Fernández, Javier Cepeda, Marta Medina O´donnell, Luis Díaz-Ruiz, Cristina Sánchez-González, Juan Llopis, José M. Seco, E. Rufino-Palomares, José Antonio Lupiáñez, Santiago Gómez-Ruiz, José L. Quiles, Maurizio Battino, Duane Choquesillo-Lazarte, Ana B. Ruiz-Muelle, Ignacio Fernández, Fernando Reyes-Zurita and Antonio Rodríguez-Diéguez

***Index:***

***1. Crystallographic tables***

***2. Stability of compounds in solution***

***Table S3. CShMs for the CoO6 coordination environment of compounds 1 and 3.***

***Figure S3. Chains constructed of mononuclear entities by hydrogen bonds in compound 1.***

***Figure S4. Chains constructed of mononuclear entities by hydrogen bonds in compound 3.***

***1. Crystallographic tables***

**Table S1.** Bond distances for *[Co(bum)_2_(H_2_O)_2_](H_2_O)_2_ (****1****).*

| **Bond Distances (**Å) |  | |
| --- | --- | --- |
| Co(1)-O(1) 2.096(3)  Co(1)-O(1)#1 2.096(3)  Co(1)-O(2) 2.236(3)  Co(1)-O(2)#1 2.236(3)  Co(1)-O(1W) 2.025(3)  Co(1)-O(1W)#1 2.025(3) | |  |
|  |  | |

**Table S2**. Hydrogen bonds for compound **1** [Å and °].

____________________________________________________________________________

D-H...A d(D-H) d(H...A) d(D...A) <(DHA)

____________________________________________________________________________

N(2)-H(2A)···O(5)#2 0.88 2.25 2.848(6) 125.0

N(2)-H(2B)···O(2W)#2 0.88 2.24 3.011(6) 145.7

O(1W)-H(1WA)···O(2)#3 0.85 1.84 2.630(5) 152.0

O(1W)-H(1WB)···O(5)#4 0.85 2.06 2.857(5) 156.0

O(2W)-H(2WA)···O(2W)#5 0.85 2.12 2.967(10) 172.2

O(2W)-H(2W)···O(1) 0.85 2.13 2.942(5) 160.3

O(2W)-H(2WB)···O(2W)#4 0.87(10) 2.04(10) 2.901(10) 173(10)

____________________________________________________________________________

Symmetry transformations used to generate equivalent atoms:

#1 -x+1,y,-z+1/2; #2 x,y-1,z; #3 -x+1,y+1,-z+1/2; #4 -x+1,-y,-z+1; #5 -x+1,-y+1,-z+1

**Table S3.** Bond distances and hydrogen bonds for *[Co(ind)_2_(EtOH)_2_] (****3****).*

| **Bond Distances (**Å) |  | |
| --- | --- | --- |
| O(1)-Co(1) 2.110(2)  O(1E)-Co(1) 2.025(2)  O(2)-Co(1) 2.185(2) | |  |
|  |  | |

***2. Stability of compounds in solution***


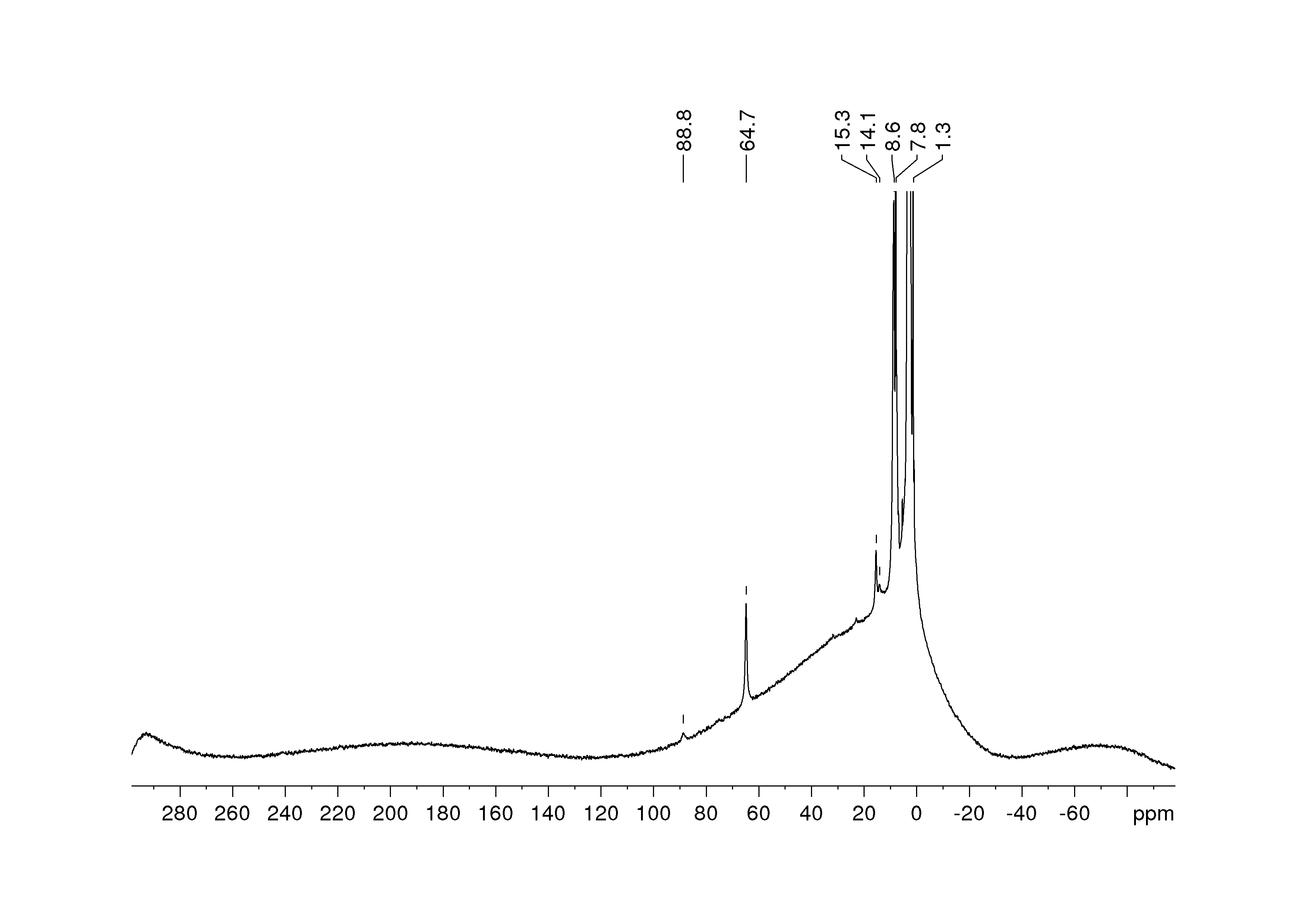


**Figure S1**. ^1^H NMR (300 MHz) spectrum of complex [Co(bum)_2_(H_2_O)_2_](H_2_O)_2_ in DMSO-*d*_6_ at 23 ºC.


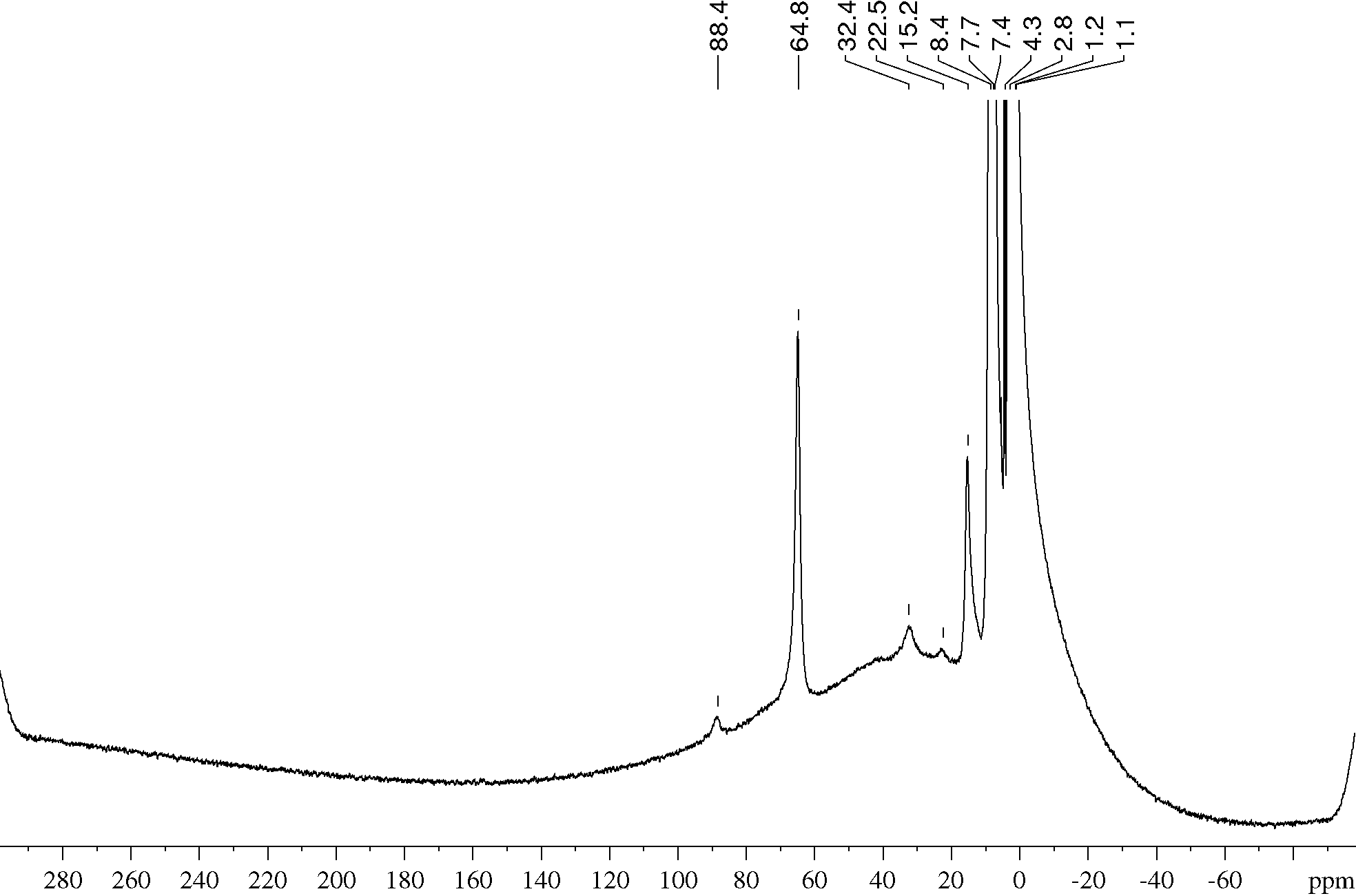


**Figure S2**. ^1^H NMR (300 MHz) spectrum of complex [Co(ind)_2_(EtOH)_2_] in DMSO-*d*_6_ at 23 ºC.

**Table S3.** CShMs for the CoO_6_ coordination environment of compounds **1** and **3**. The lowest SHAPE values for each ion are shown highlighted in grey, indicating best fits.^^[[1]](#footnote-1)^^

Codes:

HP-6 1 D6h Hexagon

PPY-6 2 C5v Pentagonal pyramid

OC-6 3 Oh Octahedron

TPR-6 4 D3h Trigonal prism

JPPY-6 5 C5v Johnson pentagonal pyramid J2

| **Structure [ML6]** | **HP-6** | **PPY-6** | **OC-6** | **TPR-6** | **JPPY-6** |
| --- | --- | --- | --- | --- | --- |
| **Comp 1** | 34.948 | 19.831 | 5.887 | 8.447 | 24.299 |
| **Comp 3** | 30.320 | 21.933 | 4.484 | 11.502 | 26.384 |


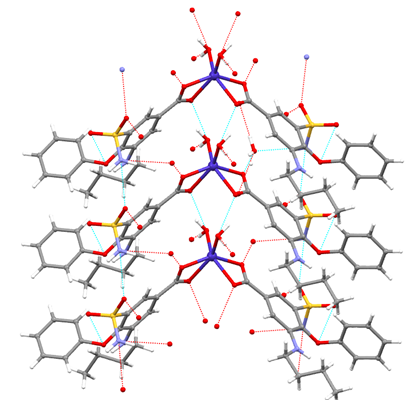


**Figure S3**. Chains constructed of mononuclear entities by hydrogen bonds in compound **1**.


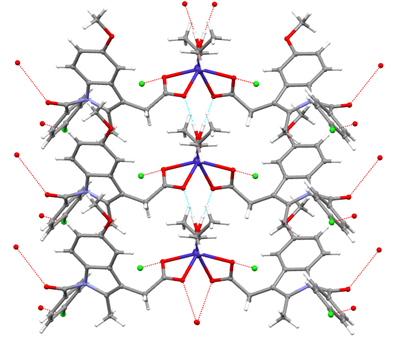


**Figure S4**. Chains constructed of mononuclear entities by hydrogen bonds in compound **3**.

1. M. Llunell, D. Casanova, J. Cirera, J. M. Bofill, P. Alemany, S. Alvarez, M. Pinsky, D. Avnir, SHAPE v1.1b, Barcelona (Spain), 2005. [↑](#footnote-ref-1)
